# From Plant Material to Environmentally Friendly Plant Growth Stimulators: Betaine-Based Ionic Liquids

**DOI:** 10.1021/acssuschemeng.5c04882

**Published:** 2025-10-13

**Authors:** Adriana Olejniczak, Michał Niemczak, Daniela Gwiazdowska, Krzysztof Juś, Andrea Mezzetta, Lorenzo Guazzelli, Damian Krystian Kaczmarek

**Affiliations:** † Faculty of Chemical Technology, 49632 Poznan University of Technology, ul. Berdychowo 4, Poznan 60-965, Poland; ‡ Department of Pharmacy, 9310 University of Pisa, via Bonanno 6, 56126 Pisa, Italy; § Department of Natural Science and Quality Assurance, Poznań University of Economics and Business, al. Niepodległości 10, Poznan 61-875, Poland

**Keywords:** ionic liquids, eco-friendly agrochemicals, thermal stability, green chemistry, ecotoxicity

## Abstract

Following global trends, the main issue is to ensure that humanity can progress without harming the environment. This article outlines the synthesis of novel ionic liquids composed of an alkylbetaine cation, whose structure is based on glycine-betaine (a byproduct of sugar beet production) and an anion based on indole-3-butyric acid (a natural auxin found in plants). The article details the synthesis method used and demonstrates its negligible environmental impact. In addition, the main physicochemical properties of ionic liquids and their initial substrates have been assessed. Biological studies indicate that these ionic liquids effectively stimulate plant growth compared to the potassium salt of indole-3-butyric acid by approximately 40–60%, promoting positive effects on plant growth after sowing, while they may also contribute to seed protection before sowing. Furthermore, environmental studies have shown that these compounds are unlikely to pose a threat to freshwater and saltwater organisms, as they fall into the Practically Nontoxic to Slightly Toxic class in the case of *A. franciscana* and the Practically Nontoxic to Moderately Toxic class in the case of *D. magna*. Moreover, ionic liquids exhibited a benign effect on soil microorganisms, with toxic effects occurring only for salts containing the longest alkyl chains (MIC = 62.5–250 μg·cm^–3^).

## Graphical abstract

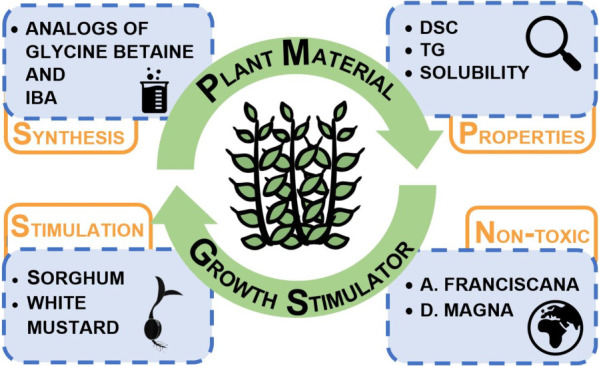

## Introduction

In recent years, studies at the intersection of chemistry and agriculture have led to breakthroughs in improving crop quality and sustainability in the field. Among these advances, ionic liquids based on indole-3-butyric acid (IBA) or glycine-betaine (GB) are promising agents in plant growth regulation (PGR).^1–3^ Ionic liquids (ILs) are widely known for their unique physicochemical properties, such as low volatility and high thermal stability, which make them extremely attractive in various scientific and industrial fields. More than two decades of intensive research unequivocally revealed that one of the most important advantages of ILs is “designability”. This means that the physicochemical properties, biological activity, and even ecotoxicity of ILs can be adjusted by the selection of appropriate cations and anions or modification of their functional groups. ILs also include bioionic liquids that are composed of cations and anions obtained from natural sources.^2^ This action facilitates the formation of biocompatible and biodegradable ILs, which are essential in the context of adhering to principles of green and sustainable chemistry.^4^ Tremendous tunability of biobased ILs also makes them ideal carriers for delivering PGRs to plants.^5^

GB is a zwitterionic compound ubiquitous in various plant species.^6^ It functions as an osmoprotectant, supporting the growth of plants exposed to abiotic stresses such as drought, salinity, and extreme temperatures.^7^ Furthermore, betaine, like auxins, contributes to modulating plant growth and development.^8^ Its derivatives, such as alkylbetaine, are an excellent substitute for synthetic cations in ILs. They not only exhibit lower environmental impact than common ILs but also possess many useful properties, e.g., surface activity,^9^ bactericidal activity,^10^ or stimulation of plant growth.^11^ Moreover, its readily biodegradable nature under aerobic conditions^12^ makes GB suitable for use as a biocarrier or as a novel source for biologically active substances demonstrating various synergistic effects.

IBA is a naturally occurring auxin that is categorized as a plant hormone.^13^ It exhibits greater stability to photodegradation compared to other auxins, therefore increasing industry interest for the preparation of formulations used in horticulture and agriculture for root-inducing capacity.^14–16^ The diversification of IBA formulations, including powders, gels, and solutions, accommodates versatile application methodologies tailored to specific requisites.^17^ Currently, ongoing scientific inquiry is dedicated to the derivatization of IBA into quaternary ammonium salts (QASs) or ILs to enhance their biological activity and physicochemical attributes.^5^

IBA and GB, together, enhance plant resilience by combining improved root growth with cellular protection, enabling better adaptation to challenging environments. In this regard, we hypothesize that combining IBA and GB within the same bio-IL may trigger synergistic effects, thus representing a substantial development in the field of PGRs. Therefore, this study aims to synthesize ILs with alkyl analogs of GB cations and IBA anion and subsequently analyze their physicochemical properties as well as biological activity. This study focused on the cations containing octyl (C_8_H_17_) or longer alkyl chains. Prior research has indicated that cations with shorter chain lengths do not possess the requisite surface activity to enhance biological activity effectively.^3^ By examining their roles in plants, particularly promoting root and shoot growth, one will be able to note the interactions of cations and anions on plant behavior. Understanding these interactions offers a pathway to developing innovative strategies for addressing the multifaceted challenges of modern agriculture, including resource efficiency and safety. In addition, to get an insight into the potential impact of these new agrochemicals on the environment, their toxicity to freshwater and saltwater crustaceans (*Daphnia magna* and *Artemia franciscana*) as well as soil microorganisms (*Prestia megaterium*, *Streptomyces violaceoruber*, *Microbacterium phyllospherae*, *Stenotrophomonas maltophilia*, *Alcaligenes faecalis*, *Fusarium graminearum*, *Pythium* sp., and *Rizoctonia solani*) was assessed. This is particularly important since these organisms can serve as key indicators of ecosystem health. These data allow us to reveal whether the designed compounds intended for use in crops pose risks or not to aquatic environments, providing a greater perspective for the development of safer and more environmentally friendly agricultural solutions.

## Materials and Methods

### Materials

Octyldimethylamine (95%), decyldimethylamine (98%), and dodecyldimethylamine (97%) were purchased from Sigma-Aldrich (St. Louis, MO, USA). Indole-3-butyric acid (98%), hydrochloric acid (36%), and chloroacetic acid (99%) were obtained from Thermo Fisher Scientific (Waltham, MA, USA). All solvents (methanol, dimethyl sulfoxide [DMSO], acetonitrile, acetone, 2-propanol, ethyl acetate, chloroform, toluene, hexane) and potassium hydroxide were delivered by Avantor (Gliwice, Poland) and used without further purification. Deionized water with a conductivity of <0.1 μS·cm^–1^ was used from the Hydrolab HLP Smart 1000 demineralizer (Straszyn, Poland). Microbiological media were used in the experiment, including Mueller-Hinton broth and TSB broth (for bacteria) and PDB broth (for filamentous fungi) purchased from BioMaxima (Poland).

### Synthesis of Alkylbetaine Indole-3-butyrate

Alkylbetaine hydrochlorides (**1**–**3**) were synthesized via the quaternization reaction of alkyldimethylamine with potassium chloroacetate and then the reaction of alkyldimethylglycine with hydrochloric acid following the methods described by Olejniczak et al.^18^ The potassium salt of indole-3-butyric acid was obtained in an analogous procedure to that previously described in the literature.^19^ The next step was to carry out an exchange of the chloride anion for the indole-3-butyrate anion. The appropriate alkylbetaine hydrochloride (0.02 mol) was dissolved in 15 cm^3^ of methanol in a reaction vessel equipped with a mechanical stirrer. Then, 0.02 mol of potassium salts of indole-3-butyric acid (pH = 7), dissolved in 15 cm^3^ of methanol, was added, and the reaction mixture was stirred at 25 °C for 10 min. Subsequently, methanol was evaporated, and then, the obtained products (**IL1**–**IL3**) were purified by leaching with a portion (15 cm^3^) of acetone to remove the traces of inorganic salts (potassium chloride, KCl). Next, the impurities were filtered off, and the solvent was evaporated from the filtrate. Finally, the obtained products (**IL1**–**IL3**) were dried at 40 °C for 72 h under reduced pressure (1–2 mbar). The reaction scheme is presented in Figure 1.

**Figure 1:**
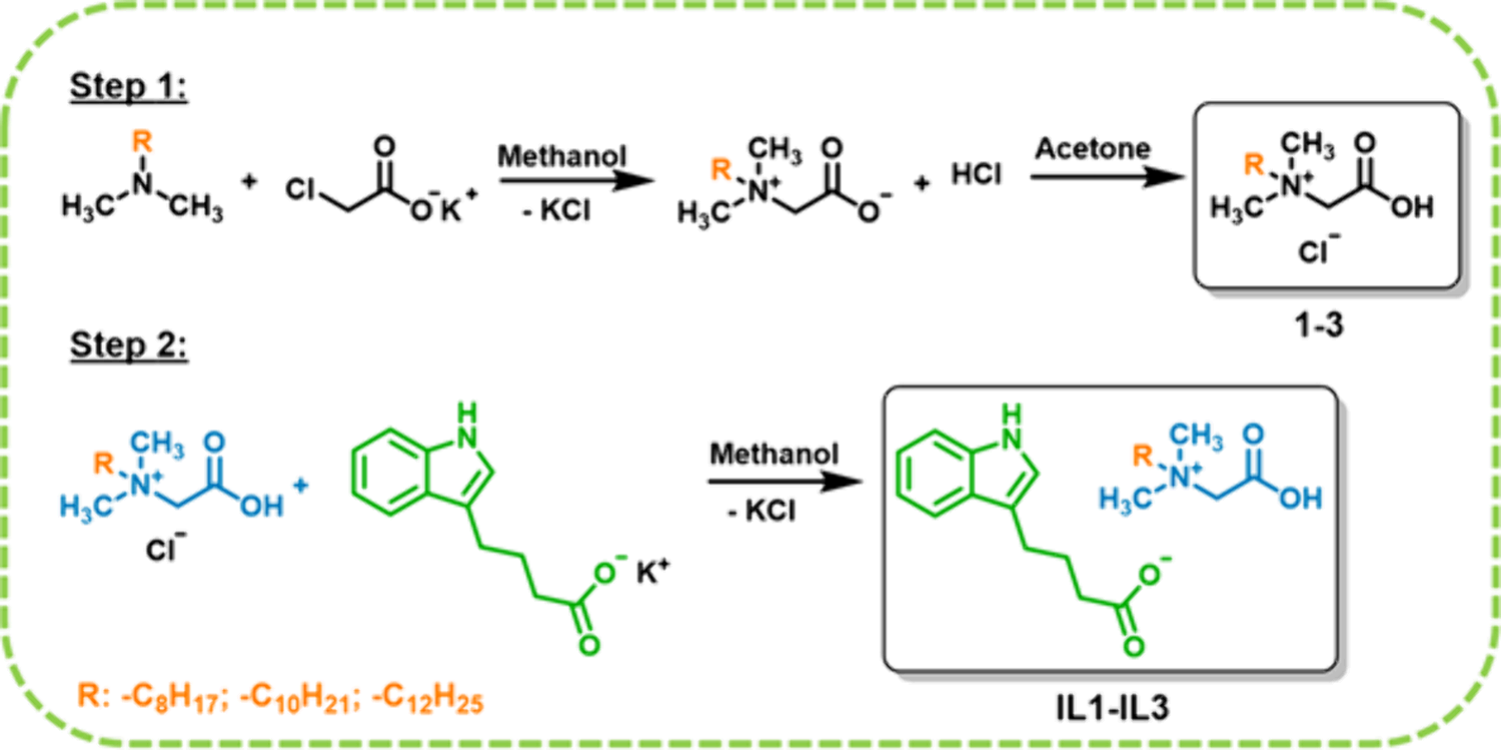
Synthesis of chloride salts **1**–**3** and ILs with indole-3-butyrate anion (**IL1**–**IL3**).

### General

Proton and carbon nuclear magnetic resonance spectra (^1^H NMR and ^13^C NMR) that confirmed the structures of the synthesized chloride salts **1**–**3** and **IL1**–**IL3** were obtained on a Varian VNMR-S spectrometer with a generation frequency of 400 MHz for ^1^H NMR and 100 MHz for ^13^C NMR. The solvent was deuterated methanol, and tetramethylsilane (TMS) was used as an internal standard.

ATR-FTIR spectra were recorded with an IR Cary 660 FTIR spectrometer (Agilent Technologies) using a macro-ATR accessory with a diamond crystal. The spectra were measured in the range from 4000 to 600 cm^–1^ with 256 scans for both background and samples.

The water content of the chloride salts and the ILs was measured with a TitroLine 7500 KF trace apparatus (SI Analytics, Germany) using the Karl Fischer titration method. First, the compound was dissolved in dehydrated methanol. The water content was assessed in pure methanol and in methanolic solutions. On the basis of the collected results, the water content in pure products was calculated.^20^

The melting points of the compounds obtained were analyzed via an MP 90 melting point system (Mettler Toledo, Switzerland). The precision of the measurements was ensured by calibrating the apparatus using certified reference substances.

The residual concentration of Cl^–^ was performed based on the method described in the literature.^21^ Obtained ILs of 1 ± 0.0001 g were introduced into a flask and mixed with deionized water to reach a volume of around 100 cm^3^. Subsequently, 1 cm^3^ of a 5% potassium chromate solution (K_2_CrO_4_) was incorporated, and the titration process commenced using a 0.1 mol·dm^–3^ silver nitrate solution (AgNO_3_) accompanied by vigorous stirring. The titration continued until a consistent brown–red suspension was achieved, signifying the conclusion of the titration. The chloride content was then computed using the following equation eq 1:

1 $$X=\frac{\left(A-B\right)\times M\times 35460}{m}$$

where

- *X* – chloride content [ppm]
- *A* – AgNO_3_ volume consumed during titration of analyzed sample [cm^3^]
- *B* – AgNO_3_ volume consumed during titration of blank [cm^3^]
- *M* – AgNO_3_ molar concentration [mol·dm^–3^]
- *m* – mass of the analyzed sample [g]

### Thermogravimetric Analysis (TGA) and Differential Scanning Calorimetry (DSC)

The thermal stability of ILs and chloride salts were investigated by thermal gravimetric analysis (TG) conducted in a TA Instruments Q500 TGA (weighing precision ±0.01%, sensitivity 0.1 μg, baseline dynamic drift <50 μg). Temperature calibration was performed using Curie point of nickel and Alumel standards, and for mass calibration, weight standards of 1 g, 500 mg, and 100 mg were used. All the standards were supplied by TA Instruments Inc. 12–15 mg of each sample was heated in a platinum crucible. First, the heating mode was set to isothermal at 60 °C in N_2_ (80 cm^3^·min^–1^) for 30 min. Then, the sample was heated from 40 to 600 °C at 10 °C·min^–1^ under nitrogen (80 cm^3^·min^–1^) and maintained at 600 °C for 3 min. Mass change was recorded as a function of temperature and time. TGA experiments were carried out in triplicate.

The thermal behavior of ILs and chloride salts were analyzed by a differential scanning calorimeter (TA DSC, Q250, USA, temperature accuracy ± 0.05 °C, temperature precision ± 0.008 °C, enthalpy precision ± 0.08%). Dry high purity N_2_ gas with a flow rate of 50 cm^3^·min^–1^ was purged through the sample. 1–5 mg of each sample was loaded in pinhole hermetic aluminum crucibles, and the phase behavior was explored under nitrogen atmosphere in the temperature range from −90 to 150 °C with a heating rate of 10 °C·min^–1^. The temperature calibration was performed by considering the heating rate dependence of the onset temperature of the melting peak of indium. The enthalpy was also calibrated using indium (melting enthalpy Δ*H*_m_ = 28.71 J·g^–1^). DSC experiments were carried out in duplicate. *T*_g_ was obtained by taking the midpoint of the heat capacity change on heating from a glass to a liquid. *T*_m_ was taken as the peak temperature of the endothermic peak on the heating run while *T*_c_ was the peak temperature of the exothermic peak on the cooling run.

### Solubility

Solubility analysis was carried out according to the method given in *Vogel’s Textbook of Practical Organic Chemistry* ^22^ with an additional test at elevated temperature. Solubility was tested in 10 solvents. Solvents used: Distilled water, Methanol, DMSO, Acetonitrile, Acetone, Isopropanol, Ethyl acetate, Chloroform, Toluene, and Hexane.

The assay was started by inserting 0.1 g of the product into a vial and then adding 1 cm^3^ of the respective solvent. The vial and its contents were stirred for approximately 1 min. A further 2 cm^3^ of solvent was added to the samples that did not dissolve, and the mixture was stirred again for 1 min. If the substance did not dissolve, the introduced modification was used and the vials were heated to 50 °C using a water bath with vigorous stirring for approximately 1 min. Based on the results obtained, the products were classified into one of three categories:

- “+”: well soluble (0.1 g per 1 cm^3^ of solvent at room temperature)
- “±”: moderately soluble (0.1 g per 3 cm^3^ of solvent at room temperature)
- “–”: insoluble (0.1 g per 3 cm^3^ of solvent at 50 °C)

### Germination and Early Development of Plants

The tests were carried out according to the methodology described in the literature based on the ISO 18763:2016 standard for assessing the effects of contaminants on germination and early plant growth.^23^ Plates, three for each test substance and three for the control sample (distilled water), were prepared. To obtain solution concentrations of 5 and 25 ppm, 0.005 or 0.025 g of IBA, respectively, were weighed into 1000 cm^3^ volumetric flasks and then topped off with distilled water. For salts with a chloride anion, the concentration was calculated to be the same cation content as that in the ILs. Subsequently, 60 g of soil was placed on each plate and mixed with 30 cm^3^ of previously prepared solutions or distilled water. The soil prepared as described above was inserted into the bottom half of the plate and covered with filter paper. At the top of the lower half of the plate, about 1 cm from the upper edge of the lower half, 10 seeds each of white mustard (*Sinapis alba* L.) and sorghum (*Sorghum saccharatum* L.) were placed.

Depending on the experimental variant, the seeds were as follows:

1. Soaked for 12 h in solutions of the test substances and then placed on soil moistened with distilled water
2. Placed without prior soaking on soil moistened with the test solutions

The plates were closed with covers and placed in an incubator at 25 ± 2 °C. Observations on plant germination were made for 4 days. After this time, each plate was photographed to measure root and shoot lengths using specialized software. Soil with the following elemental composition was used for the experiment: 67 mg P kg^–1^, 55 mg K kg^–1^, 54 mg Mg kg^–1^, 100 mg Fe kg^–1^, pH of 5.75 (in CaCl_2_), and a C organic content of 1.90% (19.00 g kg^–1^).

### Artemia fransiscana

To determine the EC_50_ parameter for the compounds, tests were carried out on marine crustaceans: *Artemia franciscana*. The methodology proposed in the Artoxkit M test (MicroBioTests Inc., Gent, Belgium) was developed according to the ASTM E1440-91 standard. The hatching process was started first. For this purpose, 50 mg of cysts was transferred to Petri dishes attached to the Artoxkit M set, which were then immersed in 10 cm^3^ of artificial seawater with salinity of 35‰ (a medium for the development of the tested organisms and a solvent for preparing solutions of the tested compounds; composition of artificial seawater: sodium chloride [26.4 g·dm^–3^], potassium chloride [0.84 g·dm^–3^], calcium chloride dihydrate [1.67 g·dm^–3^], magnesium chloride hexahydrate [4.60 g·dm^–3^], magnesium sulfate­(VI) heptahydrate [5.58 g·dm^–3^], sodium bicarbonate [0.17 g·dm^–3^], and boric acid [0.03 g·dm^–3^]) and placed at a temperature of 25 °C with access to light (6,000–10,000 lx) for 30 h. Then, 2 h before placing them on the Artoxkit M plates, 20 mg of spirulina was added to cultured Artemias. Furthermore, 1 h before analysis, the used seawater was oxygenated by passing a stream of air through the solution. To each of the 4 cells in a given row of a plate from the Artoxkit M kit, 1 cm^3^ of the solution at the specified concentrations (or the appropriate medium in the case of a control) was introduced. In the first column of cells (control sample and 5 concentrations: 0.1, 1, 10, 100, and 1000 mg·dm^–3^), no less than 30 Artemias were placed from previously prepared Petri dishes. Then, from the first cell in the first column, Artemias were collected at the selected concentration and added to the next three cells in the selected row with 10 organisms per cell. After the organisms were introduced into the appropriate cells, the plates were covered with parafilm and then closed with a plastic cap. The prepared kit was incubated at 25 °C and protected from light. The number of motionless organisms were counted after 24 and 48 h. Immobilization was then calculated (eq 2) in relation to the number of organisms at the start of the test:

$$\%\;immobilization=100\cdot \left(1-\frac{no.\;of\;mobile\;organisms\;after\;24\;or\;48\;h}{initia\,l\;no.\;of\;mobile\;organisms}\right)$$ 2

### Daphnia magna

The plates for the tests were purchased from MicroBioTests Inc. (Gent, Belgium), while medium and cultured freshwater organisms, ready for analysis, were prepared according to the OECD 202 guideline. The medium for preparing solutions of the analyzed substances, as in the case of *A. franciscana*, was oxygenated 1 h before the tests, and the organisms were fed with spirulina 2 h before the start of the test. To each of the 5 cells in a given row from the Daphtoxkit F kit, 10 cm^3^ of the solution at the specified concentrations (or the appropriate medium in the case of a control) was introduced. Next, more than 20 Daphnia were placed in the first column of the cells (control and 5 concentrations: 0.1, 1, 10, 100, and 1000 mg·dm^–3^). Then, from the first cell in the first column, 5 Daphnia were taken at the selected concentration and added to the next four cells in the selected row. After the organisms were introduced into the appropriate cells, the plate was covered with parafilm and then closed with a plastic cap. The prepared kits were incubated at 25 °C and protected from light. Mortality results were collected after 24 and 48 h. The immobilization and EC were calculated analogously as in the case of Artemia (eq 2).

### Microbial Toxicity Assay

#### Microorganisms

The effect of the obtained compounds on the growth of soil microorganisms was determined towards Gram-positive bacteria (*Prestia megaterium*, *Streptomyces violaceoruber*, and *Microbacterium phyllospherae*), Gram-negative bacteria (*Stenotrophomonas maltophilia*, and *Alcaligenes faecalis*), and filamentous fungi (*Fusarium graminearum*, *Pythium* sp. and *Rizoctonia solani*). Bacterial strains were isolated from soil, identified by the MALDI-TOF MS method, and deposited in the collection of the Department of Natural Science and Quality Assurance, Poznań University of Economics and Business. Fungal strains were obtained from the collection of the Research Centre for Registration of Agrochemicals and the Bank of Plant Pathogens, Institute of Plant Protection – National Research Institute in Poznań, Poland.

#### Determination of MIC and MBC/MFC

Minimal inhibitory concentration (MIC) and minimal bactericidal/fungicidal concentration (MBC/MFC) of the obtained compounds were determined using the microdilution method based on methodology described by Gwiazdowska et al.^24^ with some modifications. First, a series of 2-fold dilutions of the obtained compounds in the concentration range of 0.50–1000 μg·cm^–3^ was prepared in 96-well microplates in MHB:TSB (1:1, v:v) for bacteria as well as PDB for filamentous fungi. Then, from fresh cultures of indicator microorganisms, suspensions were prepared in broth media at a final concentration of 10^5^ CFU·cm^–3^ bacterial cells and 10^6^ conidia·cm^–3^ fungal cells, which were inoculated into the prepared microplates. Incubation was carried out for 24 h and 5–7 days at 25 or 30 °C, depending on the indicator microorganism. After incubation, the optical density of bacteria growth was determined at 600 nm using a BioTek Epoch 2 microplate reader, while in the case of fungi, mycelium growth in the microplate wells was visually observed. The results are expressed as the average of three replicates. The MIC value was defined as the concentration of tested substances, inhibiting the growth of the bateria by at least 90% whereas 100% of inhibition was defined as minimum bactericidal concentration (MBC). In the case of fungi, only concentrations at which a complete lack of mycelium development was observed were taken into account (MIC = MFC).

## Results and Discussion

### Synthesis and Structure Confirmation

Novel ionic liquids were obtained using natural origin reagents or their simple analogs, such as GB derivatives (a sugar beet product) and IBA (a plant hormone), by “green” synthesis at room temperature using methanol or acetone as solvents. Their use is highly recommended as a more environmentally-friendly alternative to chlorinated solvents, ethers, or dimethylformamide (DMF),^25^ which are widely utilized in the synthesis protocols of multiple ILs. It should also be highlighted that the selection of three alkylbetaine salts with alkyl chains containing 8, 10, or 12 carbon atoms was motivated by environmental aspects, where their minor or moderate toxicity was previously reported.^12,26^ Drawing inspiration from the growth-promoting properties of GB^27^ and IBA,^28^ we have combined alkylbetaine hydrochlorides with potassium salts of IBA to formulate novel plant growth stimulators. Table 1 illustrates the yield, water content, and chloride content of the salts obtained.

**Table 1:** Synthesized Chloride Salts (**1**–**3**) and ILs with an Indole-3-butyrate Anion (**IL1**–**IL3**)

No.	State at 25 °C	Yield [%]	Water content [%]	Cl^–^ content [ppm]
**1**	solid	96	0.20	
**2**	solid	97	0.20	
**3**	solid	98	0.13	
**IL1**	liquid	96	2.16	651
**IL2**	liquid	95	2.40	760
**IL3**	liquid	97	0.76	1200

The data revealed that **IL1**–**IL3** exhibited a higher water content than chloride salts **1**–**3**. This observation can be explained by stronger hydrogen bonding interactions between water molecules and the obtained ILs, which requires a significantly greater amount of energy for removal of water molecules from the existing H-bond networks. Furthermore, it is not feasible to reduce the water content below certain thresholds without highly specific equipment, like a sealed glovebox for work in an inert gas atmosphere with an atmosphere drying module.^29^ Additionally, an increase in water adsorption from the atmosphere was noted for **IL1**–**IL3**, resulting in a higher overall water content than that observed in the analyzed chloride salts **1**–**3**. This effect was confirmed by repeating the water content analysis for compounds exposed to ambient conditions. Moreover, chain elongation greater than ten carbon atoms contributes to notably enhanced hydrophobicity; thus, for **IL3** and chloride salt **3**, the water content was found to be the lowest. The chloride anion content ranged between 650 and 1200 ppm clearly indicating the presence of a very small amount of impurities. Nonetheless, the content of both Cl^–^ and H_2_O should not pose any serious issues regarding the application of these salts as PGRs. While the measured chloride content is unlikely to pose any hazard to either plant health or environmental safety,^30^ it remains essential to consider the water content when preparing suitable concentrations for bioassays and subsequent commercial formulations.

FTIR spectra, along with the ^1^H and ^13^C NMR spectra, were collected for the structures of chloride salts **1**–**3** and **IL1**–**IL3**. All spectra are presented in the Figures S.1–S.18. Characteristic absorption bands were identified in the FTIR spectra (chloride salts **1**–**3**: alkyl C–H stretching approximately 2950–2850 cm^–1^, carboxylic C=O stretching approximately 1733 cm^–1^, carboxylic C–O stretching approximately 1195 cm^–1^; **IL1**–**IL3**: for indole, anion N–H stretching approximately 3240 cm^–1^, aromatic C–H stretching approximately 3050 cm^–1^, carboxylate C =O stretching approximately 1630 cm^–1^, and out of plane bending of aromatic C–H approximately 740 cm^–1^, for alkyl betaine, the alkyl C–H stretching approximately 2922–2853 cm^–1^ and carboxylic C=O stretching approximately 1706 cm^–1^), along with signals in the NMR spectra at corresponding chemical shifts (indole ring: approximately 6.90–7.60 ppm on ^1^H NMR spectra and approximately 112.2–138.1 ppm on ^13^C NMR spectra; carboxymethyl group in cation: approximately 3.70–3.71 ppm on ^1^H NMR spectra and 168.7 ppm on ^13^C NMR spectra). This allowed for the confirmation of the desired structures. The characteristic bands from FTIR spectra and chemical shifts from NMR spectra have been summarized in Tables 2 and 3. These data allow one to observe the differences between the ILs (**IL1**–**IL3**), the chloride salts (**1**–**3**), and IBA.

**Table 2:** Summary of Selected FTIR Data of **IL1**–**IL3**, Chloride Salts **1**–**3**, and IBA

	Wavelength (cm^–1^)		
Functional group	**IL1**–**IL3**	Salts **1**–**3**	IBA^31^
C=O (IBA)	1630		1695
C=O (cation)	1706	1733	
N–H (IBA)	3240		3392

**Table 3:** Summary of Selected NMR Data of **IL1**–**IL3**, Chloride Salts **1**–**3**, and IBA

	Chemical shift (ppm)				
	^1^H NMR			^13^C NMR	
Functional group	**IL1**–**IL3**	Salts **1**–**3**	IBA^2^	**IL1**–**IL3**	Salts **1**–**3**
CH_2_–COO (IBA)	2.33		2.43	178.0–178.3	
CH_2_–COO (cation)	4.32–4.35	3.70–3.71		168.7	167.3
C_*n*_H_2*n*+1_–CH_2_–N+ (cation)	3.59–3.60	3.38–3.41		65.5	66.6
CH_3_–N+ (cation)	3.30–3.32	3.08–3.09		50.5	52.2

### Green Chemistry Metrics

*Green Chemistry* metrics, e.g., atom economy, percentage yield, environmental factor, and reaction mass efficiency, are important parameters used to assess the environmental impact of chemical processes and products. Focusing on key factors such as atom economy or waste reduction, they provide a standardized framework for assessing the overall impact of chemical practices.^32^ Therefore, *Green Chemistry* metrics were determined for chloride salts **1**–**3** and **IL1**–**IL3**, and the results are presented in Figure 2 (specific data are shown in Table S1).

**Figure 2:**
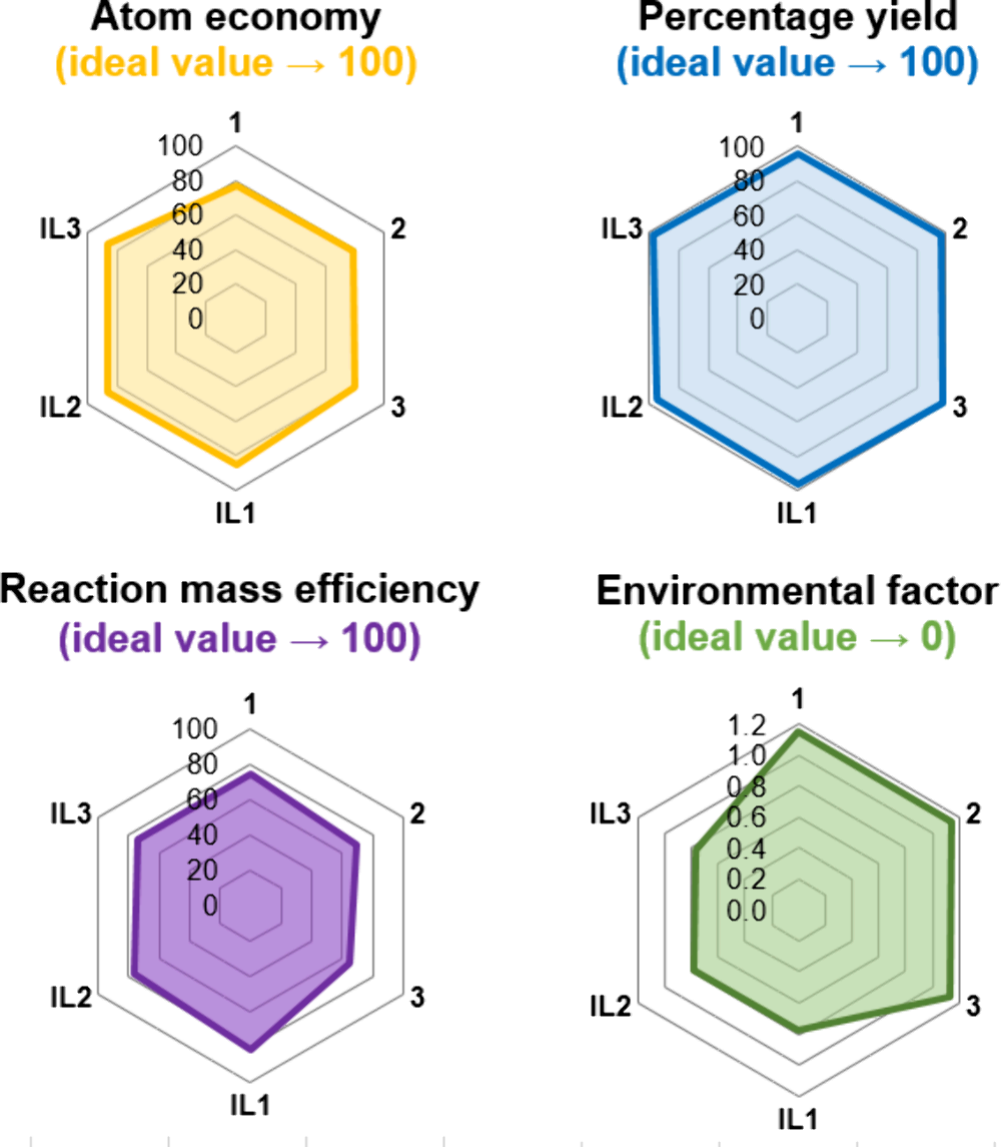
Radar plot of Green Chemistry metrics determined for chloride salts **1**–**3** and ILs (**IL1**–**IL3**).

Atom Economy (ideal value of 100%) and Reaction Mass Efficiency (ideal value of 100%) outcomes highlighted the high utilization of reactants in the synthesis and confirmed that the amount of waste is relatively low. These parameters also demonstrated that there was more waste produced in the first step of the synthesis than in the second one. Yields were almost quantitative for all compounds (chloride salts **1**–**3** were obtained with reaction yields above 96%, while **IL1**–**IL3** had reaction yields above 95%). Overall, apart from a small amount of unreacted substrates, the main waste produced was potassium chloride. It is worth mentioning that, from the industrial perspective, this reaction waste may be reintroduced in the value chain by using it directly in agricultural applications or alternatively for the synthesis of potassium hydroxide.^33^

Among the many important parameters used to assess the environmental sustainability of a synthetic pathway, the Environmental Factor (E-factor) is frequently considered the most essential. This parameter was equal to 1.14 for the first step of the synthesis and 0.77 for the second step. Nonetheless, based on Roger A. Sheldon’s data, even the first stage of synthesis agrees with the lower limit accepted for the bulk chemicals industry.^32^ Thus, the synthesis of the ILs is below the assumed threshold for application in industry. Furthermore, the complete synthesis was also considered, where the E factor for the synthesis of all ILs was estimated as 2.5, which is still in line with the bulk chemicals production.^32^

### Analysis of Thermal Properties and Solubility

The thermal behavior obtained by DSC along with the thermal stability analyzed by TGA and literature data are summarized in Table 4 (DSC and TGA thermograms are shown in Figures S.19–S.30). The results of the solubility tested in water and in a variety of organic solvents are presented in Table 5.

**Table 4:** Phase Transitions and Thermal Stability for Chloride Salts **1**–**3** and **IL1**–**IL3**

	*T*_m_ [°C]					
No.	This study	Literature	*T*_c_ [°C]	*T*_g_ [°C]	*T*_onset_ [°C]	*T*_5%_ [°C]
**1**	104	161–163 ^34^	95		197	185
**2**	110	152–158 ^18^	96		195	182
**3**	117	158–160 ^18^	86/103		201	187
**IL1**				–15	236	226
**IL2**				–14	232	222
**IL3**				–13	241	234

**Table 5:**
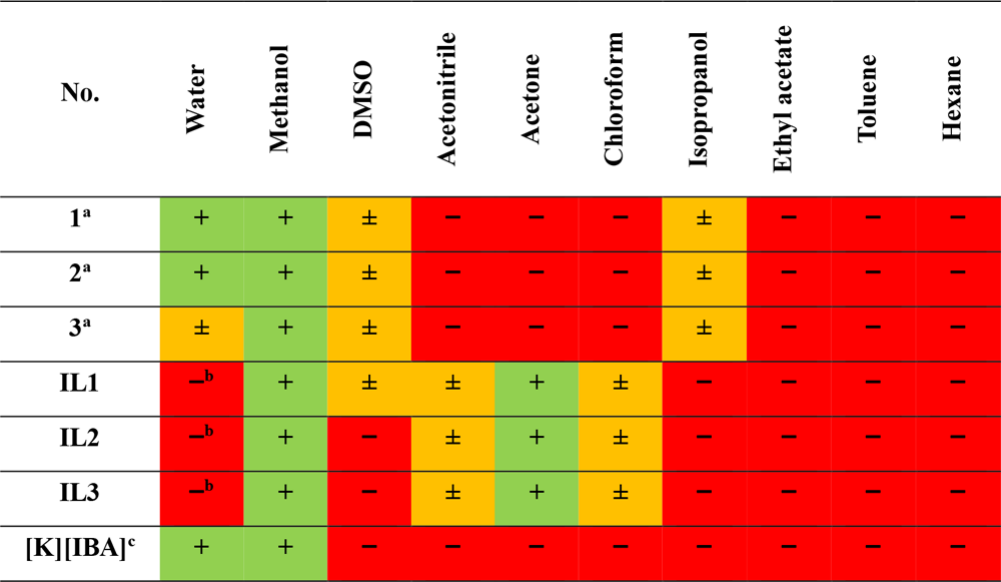
Solubility of chloride salts **1**–**3** and **IL1**–**IL3**

a Literature data.^18^

b 7.5 g of the compound is dissolved in 1000 cm^3^ of water.

c Potassium salt of indole-3-butyric acid.

The melting point (*T*_m_) obtained by DSC revealed substantial dissimilarities when compared with those recorded by visual inspection (MP 90 melting point system). The DSC analysis indicated that the chloride salts **1**–**3** melted within the range of 104–117 °C, whereas literature values suggest they should melt approximately between 150 and 165 °C. This discrepancy prompted an investigation using the Melting Point System MP-90, which allows for the analysis of recorded video footage. By examination of the video frame by frame between 100 and 120 °C, it was observed that the compounds did not convert into a transparent liquid but instead formed a white-colored wax. Notably, discoloration and reduced viscosity of the substance occurred around 160 °C, coinciding with the onset of compound degradation (see Figure 3). After careful analysis, these data suggest that methods based on visual assessment can be greatly inaccurate as the method of analysis is highly subjective. As was demonstrated above, visual methods may prove to be particularly inadequate for compounds that do not transform into transparent liquids. Moreover, chloride salts **1**–**3** showed a crystallization temperature (*T*_c_) at approximately 100 °C. Chloride salt **3**, however, is an exception as it exhibited two *T*_c_ (86 and 103 °C). This phenomenon may be attributed to the polymorphic nature of cations with long alkyl chains, which can form two distinct crystalline systems in a single cooling cycle.^35^ The initial crystallization could involve the metastable phase at a higher temperature, while the subsequent crystallization could involve the more stable phase at a lower temperature. Conversely, **IL1**–**IL3** displayed only a glass transition (*T*_g_) at approximately -13 to -15 °C.

**Figure 3:**
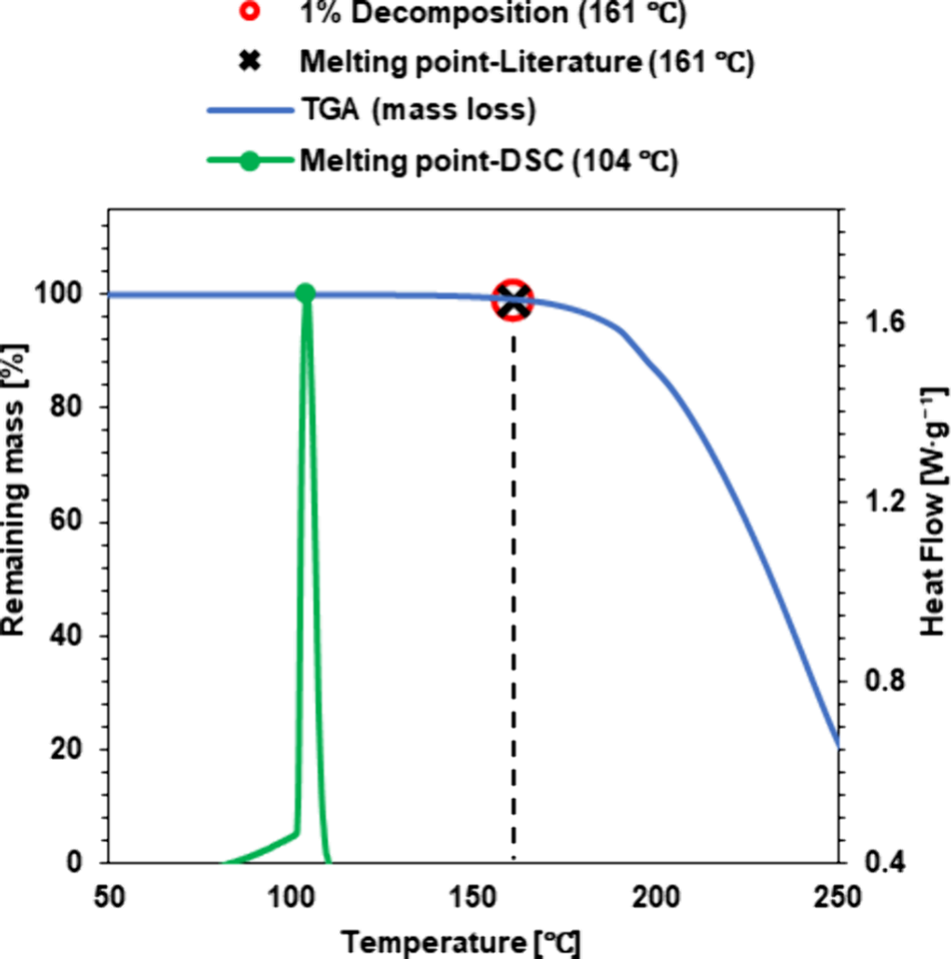
Comparison of melting point (DSC analysis and visual observation, literature) and decomposition temperature for chloride salt **1**.

The solubility analysis of both salts with the chloride anion (**1**–**3**) and the IBA anion (**IL1**–**IL3**) indicated that the combination of two compounds with very good or good water solubility contributed to the formation of a new compound with reduced solubility. The explanation for this phenomenon is to be found in the difference in charge density and bonds formed between the cation and the anion, as described by Hurst and Fortenberry.^36^ Furthermore, a reduction in solubility was noted during dissolution in 2-propanol and DSMO, and the opposite effect was observed for acetonitrile, acetone, and chloroform. For the other solvents, the solubilities for the substrate and the salt were the same. An important supplement to the basic solubility analysis was the **IL1**–**IL3** fine dissolution test in water. This was necessary to determine the feasibility of further bioassays. A solubility of 7.5 g·dm^–3^ was sufficient to prepare suitable aqueous solutions for further studies. Additionally, the water solubility of the pure active ingredients of IBA or **[K]­[IBA]** contained in commercial products was compared with that of **IL1**–**IL3**. The solubility of **IL1**–**IL3** improved 30-fold compared to IBA,^5^ while it decreased 13-fold compared to **[K]­[IBA]**. Compared to previously described ionic liquids, ammonium salts, and binary mixtures containing IBA, the synthesized **IL1**–**IL3** exhibited a 3- to 4-fold decrease in solubility.^2,3,5^ This difference can be attributed to variations in bonding types, molecular interactions, or structure. The solubility differences were determined by the amount of compound dissolved in a constant volume of water. Nevertheless, the reduced solubility of ILs is not an issue compared to the concentrations used in commercial formulations.^37^ Another advantage of **IL1**–**IL3** is that their aqueous solutions can reduce surface tension due to the cation exhibiting this property.^38^ It is worth highlighting that, below the solubility threshold, the **IL1**–**IL3** emulsions foamed slightly. Thus, **IL1**–**IL3** improved solubility compared to IBA.

### Germination and Early Development of Plants

When examining the effects of novel chemicals on seed germination and early development of plants, it is essential to assess not only their efficacy as agrochemicals (e.g., herbicides, fungicides, PGRs) but also their toxic effects on the environment (agricultural soil contamination). White mustard and sorghum are model organisms in this assay due to their quick growth rates and sensitivity to various chemical agents.^23^ These species are also used in studies that have demonstrated their responsiveness to IBA in stimulating root development and growth.^2,39^ Noteworthy, both species are agronomically relevant crops with well-documented physiological responses, which makes them suitable indicators of bioactivity for auxin-related compounds. Their contrasting botanical characteristics (dicotyledonous, white mustard, and monocotyledonous, sorghum) also allow evaluation of compound effects across different plant types, providing broader insights into the potential applicability of newly synthesized derivatives. Moreover, based on their common use in agriculture, the results of these studies can provide valuable information on practical applications in agrochemicals. Two different studies were conducted. In the first case, aqueous solutions of the studied substances were applied to the soil. Conversely, in the second case, seeds were soaked in aqueous solutions of the studied substances for 12 h before sowing into the water-soaked soil.

In the first study, it was determined whether the injection of 5 and 25 ppm of IBA aqueous solutions (for the chloride salts **1**–**3**, the concentration converted to the same cation content as in **IL1**–**IL3**) into the soil would lead to a modification in the germination or length of the roots and shoots of white mustard and sorghum. The results showed that neither inhibitory nor stimulatory effects occurred: all plants developed similarly to the control. Therefore, it can be concluded that chloride salts **1**–**3** and **IL1**–**IL3** do not exhibit any stimulating or toxic effects at these concentrations. This indicates that neither the cation nor the anion affects white mustard and sorghum at the studied concentrations. Nevertheless, it is worth noting that the concentrations impacting plant development can vary depending on the species. For instance, in maize treated with ILs containing 5 ppm of IBA, it was observed that the roots were statistically longer than for the control when these substances were introduced into the soil.^5^

In the second study, analyses were carried out to determine the effect of soaking white mustard and sorghum seeds in aqueous solutions with an IBA concentration of 25 ppm for 12 h before sowing (control seeds soaked in distilled water; for chloride salts **1**–**3**, the same cation concentration as for **IL1**–**IL3** was used). Following, the seeds were transferred to plates with distilled water-soaked soil. The obtained results, presented in Figure 4, proved that IBA in the form of potassium salt, along with the synthesized ILs, can exert a strong influence on early plant development. When the seeds were soaked with aqueous solutions of products at a concentration of 25 ppm IBA, the stimulating effect on a root or shoot growth was noted in the case of white mustard, whereas on sorghum only shoot length was enhanced. It should be emphasized that, in the sorghum trials, **IL1** was the only one that additionally stimulated sorghum root growth. The influence of chloride salts **1**–**3** on plant development was found to also be deprived of usual tendency. While the chain length in the cation consisting of 10 or 12 carbon atoms (chloride salts **2** and **3**) was inert to the plants, chloride salt **1** with 8 carbon atoms in the chain exhibited mainly inhibitory and detrimental effects on their growth. However, it was also noticed that the substitution of the chloride anion with IBA results in the strongest stimulating effect on the growth of both plants. A possible explanation of such excellent activity shown by **IL1** may be related to the cation that contains 8 carbon atoms. This can more freely permeate through plant membranes since it is highly soluble in water and has a good affinity for hydrophobic surfaces. Thus, combining such a cation (with some plant growth inhibitory properties) with an IBA anion, which is a natural auxin, contributes to negation of the toxic effect of the cation, which serves mainly as a carrier for the active substance. It is also noteworthy that the elongation of the alkyl chain in the cation causes a less pronounced biological effect: chloride salts **2** and **3** are inert, while **IL2** and **IL3** stimulate plant growth to a lower extent. This trend can be explained by the compounds’ greater molar volume and hydrophobicity, leading in consequence to the longer transport time of the active substance within the plants.^40^ In previous studies, a combination of GB and IBA was described and tested as a natural plant stimulator. The study illustrated that such a mixture stimulates the root of white mustard growth at a concentration of 25 ppm, but shoots are statistically similar to the control.^2^ Comparing these data with the results for **IL1**–**IL3**, it was noted that the ILs stimulate the growth of both the roots and shoots of the plant. Therefore, the stimulating effect was stronger for **IL1**–**IL3** synthesized than for a mixture of IBA and GB.

**Figure 4:**
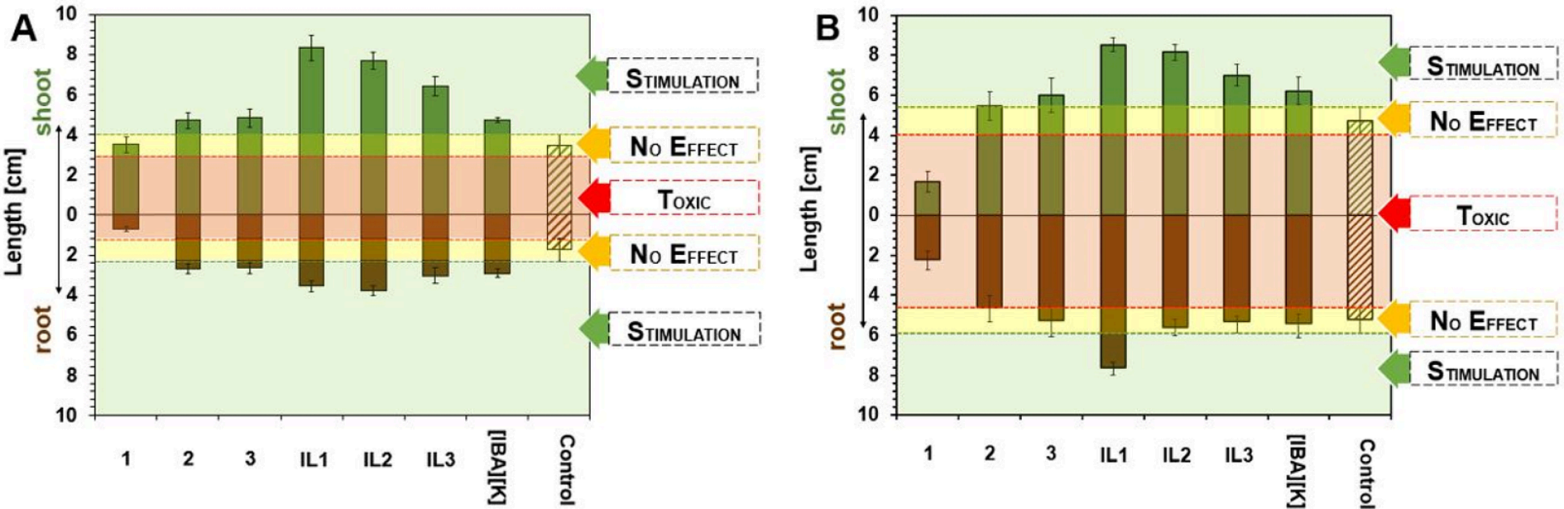
Effect of 25 ppm of IBA aqueous solutions of the ILs and the chloride salts on the shoot and root length of white mustard (A) and sorghum (B). Control, control sample without the addition of chloride salts or ILs.

These studies highlight a crucial aspect of plant research: analysis of the stimulating or inhibiting effect on plant growth due to the application of an active substance is a complex process. For instance, it is essential to consider various additional variables, such as consistent humidity, temperature, and timing, that must be kept under control. Furthermore, the concentration of the active substance, the method of its application, and the plant growth stage at which it is introduced are critical factors that significantly influence the final results. Consequently, it can be concluded that the treatment of seeds with the ILs at concentrations below 25 ppm should not have a negative effect on their subsequent growth and even stimulate root and shoot growth. Furthermore, a deeper analysis of the knowledge about ILs and alkylbetaine cations reveals the promising potential of **IL1**–**IL3**. The figures on the bactericidal and fungicidal properties of the cation^10^ suggest that the ILs could fulfill as protectants for seeds, preserving them from microbial damage before sowing. Nevertheless, further studies are essential to validating this hypothesis. These studies will help establish the role of **IL1**–**IL3** as multifunctional compounds in agricultural applications.

### Aquatic Toxicity toward Crustaceans

Crustaceans such as *D. magna* and *A. franciscana* play a key role in aquatic ecosystems, serving as an important link in food chains and providing a major food source for many species of fish and other predators. Their sensitivity to toxic substances makes them excellent indicators of ecotoxicity, ultimately allowing one to assess the impact of chemicals on the entire aquatic ecosystem and, consequently, biodiversity. Numerous organizations involved in establishing global standards and regulations for environmental protection, public health, and sustainable development consider the EC_50_ range or its specific value in crustaceans to be a significant indicator of the impact of a substance on aquatic systems. These model organisms enable rapid evaluations, providing early insights into both environmental safety and the efficacy of compounds against plant pathogens. Furthermore, the simplicity of these tests in various cases minimizes the need for testing higher organisms, thereby supporting ethical and cost-effective research practices, while offering early warnings of potential threats to aquatic ecosystems. The distinction between the two crustaceans lies in their habitats: Artemia exists in brackish water, whereas Daphnia inhabits freshwater environments. This particular difference means that two species had to adapt to different aqueous ecosystems; hence, they are considered excellent model organisms for testing novel agrochemicals. In consequence, ecotoxicity assessment performed toward both Artemia and Daphnia provides a wide insight into the potential thread of synthesized compounds toward aqueous environments including rivers, lakes, and seas.

#### *Artemia franciscana*: Marine Crustacean

The ecotoxicity studies performed with *A. franciscana* revealed that the investigated compounds do not pose a severe threat to marine organisms regardless of the length of the alkyl chain in the cation (Table 6). The EC_50_ values of most of the substances tested (the chloride salts **1** and **2** as well as **IL1** and **IL2**) are in the range of 100–1000 mg·dm^–3^ and, therefore, fall within the “Practically Nontoxic” range, suggesting that they have little effect on *A. franciscana*. **3** and **IL3** show EC_50_ values between 10 and 100 mg·dm^–3^ for both 24 and 48 h exposure periods, classifying them as “Slightly Toxic”. This increased toxicity compared to the other substances tested may result from their distinct chemical properties, such as higher lipophilicity, which facilitates their accumulation in aquatic organisms and enhances toxicity over a short exposure period. For **[K]­[IBA]**, a shift in EC_50_ values is observed between 24 h (>1000 mg·dm^–3^) and 48 h (100–1000 mg·dm^–3^), confirming its benign nature at shorter exposure times. Nevertheless, this suggests that the substance may exhibit increased bioavailability to the organism over time or that its accumulation contributes to enhanced toxicological effects. It should also be noted that **[K]­[IBA]** was found to be less toxic than **IL3**, indicating that its toxicity was significantly influenced by the length of the alkyl chain present in its structure and not by the anion itself.

**Table 6:**
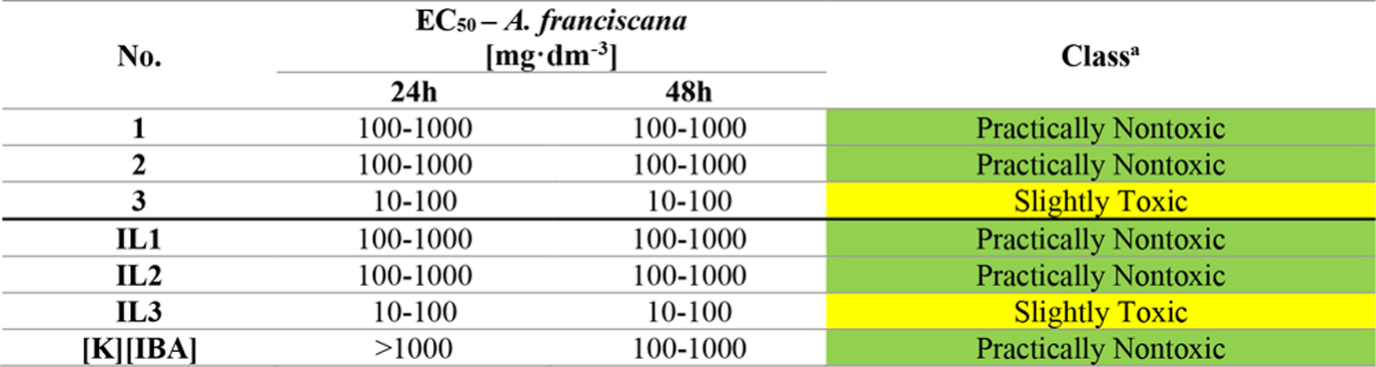
Ecotoxicity toward *A. franciscana* for Analyzed Compounds

a After 48 h. Classes were assessed according to the acute toxicity rating scale by Fish and Wildlife Service.

It is important to note that most QASs have been identified as highly toxic to aquatic organisms; this is frequently considered as their primary drawback for commercial applications. Fascinatingly, structurally similar betaine alkyl esters, containing 8, 10, and 12 carbons^41^ in the alkyl chain and a bromide anion, showed greater toxicity to brackish water crustaceans. These analogs were classified as “Slightly Toxic” to “Moderately Toxic”, underscoring the pivotal influence of anion type and the location of alkyl chain on marine organisms, like *A. franciscana*. Moreover, other betaine-type ILs precursors in their zwitterionic form were classified as “Practically Nontoxic” (EC_50_ of 100–1000 mg·dm^–3^), highlighting the profound impact that different molecular forms of a single compound can exhibit on such a sensitive ecosystem.^42^

#### *Daphnia magna*: Freshwater Crustacean

The EC_50_ ranges collected for *D. magna* highlighted diverse levels of toxicity among the tested substances (Table 7). Chloride salts **2** and **3** as well as **IL2** and **IL3** exhibited EC_50_ values in the range of 1–10 mg·dm^–3^ after 24 h as well as 48 h, categorizing them as “Moderately Toxic”. Notably, **IL1** displayed significantly lower toxicity (EC_50_ of 100–1000 mg·dm^–3^) compared to its precursor (**1**) (EC_50_ of 10–100 mg·dm^–3^), suggesting a potential mitigating effect of the IBA ion on the ionic pair overall toxicity toward *D. magna*. Similarly, **[K]­[IBA]** showed the lowest toxicity with EC_50_ values within the 100–1000 mg·dm^–3^ range at 48 h, classifying it as “Practically Nontoxic”. Literature data indicate that IBA has an EC_50_ of approximately 57 mg·dm^–3^ for *D. magna*, classifying it as the “Slightly Toxic” compound.^43^ IBA showed greater toxicity to *D. magna* than **IL1** and **[K]­[IBA]**, but it was less toxic than **IL2** and **IL3**. These findings emphasize the significant influence of the cation structure in ILs on toxicity toward *D. magna*, which is plausibly due to differences in the chemical properties and interactions between specific ions. Furthermore, the tests performed on *D. magna* clearly indicate that an increase in the alkyl chain length in the tested ILs, similarly to *A. franciscana*, contributes to increased potential ecotoxic effects.

**Table 7:**
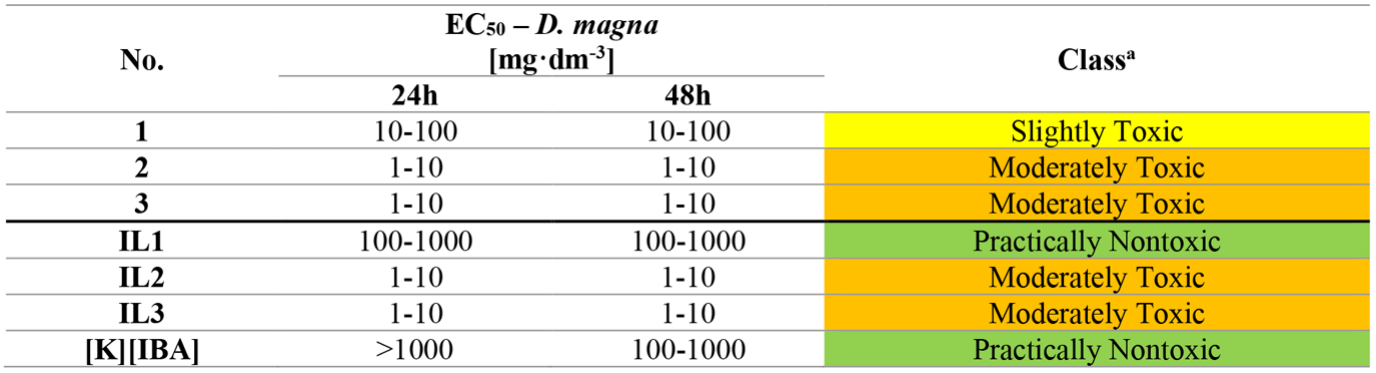
Ecotoxicity toward *D. magna* for Analyzed Compounds

a After 48 h. Classes were assessed according to the acute toxicity rating scale by Fish and Wildlife Service.

Interestingly, other ILs comprising a betaine ester with a short alkyl chain (two carbon atoms) cation and a levulinate anion showed low toxicity toward freshwater crustaceans, qualifying them in the “Practically Nontoxic” range.^44^ This confirms the widely known trend in which the hydrophobicity of a compound, conferred by the elongation of the carbon chain, simultaneously increases the toxicity of the compound. Previously reported betaine alkyl esters with alkyl chains containing 8, 10, and 12 carbon atoms and a bromide anion were classified as “Slightly Toxic” to “Moderately Toxic” for *D. magna* and *A. franciscana*.^41^ Conversely, for chloride salts **1**–**3** and **IL1**–**IL3**, no clear relationships between crustaceans’ sensitivity and the structures were observed. Therefore, the determining factor in this case may be the IBA anion or its specific interactions with betaine-type cations.

### Toxicity towards Soil Microorganisms

Testing the impact of agrochemicals on soil microorganisms is crucial for soil health and fertility, as well as microbial diversity and ecosystem balance. The analysis of toxicity to the soil microbiome is equally important due to the biodegradability of these substances: the lack of microorganisms in the soil means that the decomposition process is inhibited and the soil is contaminated.^45,46^ The toxicity of chloride salt **1**–**3**, **IL1**–**IL3**, and **[K]­[IBA]** toward microorganisms isolated and identified from agricultural soil where maize was cultivated, including Gram-positive and Gram-negative bacteria, as well as fungi, are presented in Table 8.

**Table 8:**
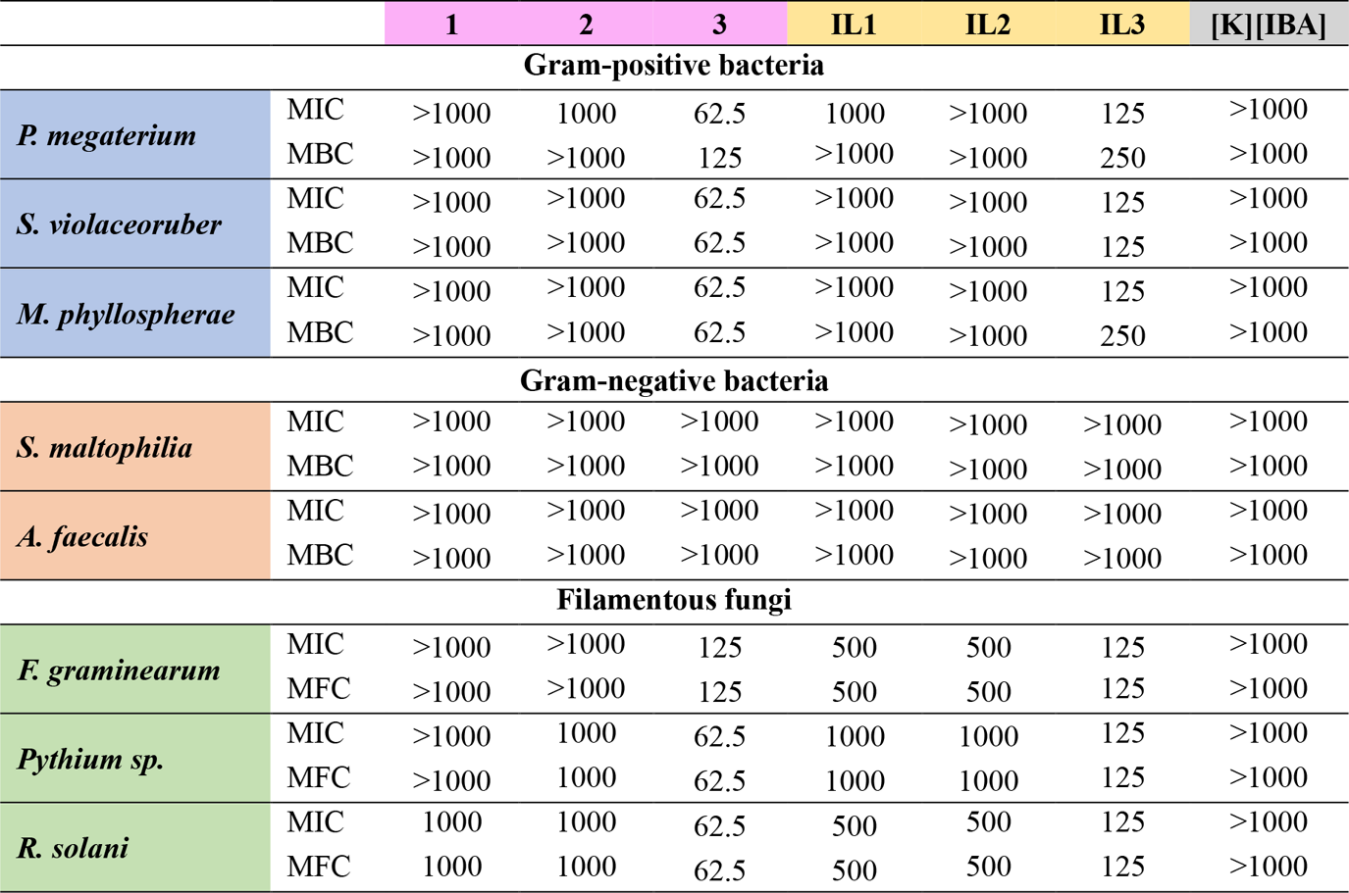
MIC (μg·cm^–3^) and MBC or MFC (μg·cm^–3^) Determined for Chloride Salts **1**–**3**, **IL1**–**IL3**, and **[K]­[IBA]**

The obtained results indicated that none of the examined compounds exhibited inhibitory activity toward Gram-negative bacteria. This outcome is consistent with the well-documented inherent resistance of these microorganisms, which is attributable to the presence of an outer membrane that functions as an effective permeability barrier.^47^ In contrast, the toxicity of the tested compounds toward Gram-positive bacteria and fungi varied.

Chloride salts **1** and **2** indicated no significant microbial toxicity. Conversely, **IL1** and **IL2** exhibited weak antifungal activity, inhibiting the growth of *F. graminearum* and *R. solani* with an MIC of 500 μg·cm^–3^, while showing no detrimental effects on Gram-positive bacteria. The most pronounced antimicrobial effects were observed for chloride salts **3** and **IL3**. Chloride salt **3** inhibited the growth of Gram-positive bacteria and fungi with MIC values ranging from 62.5 to 125 μg·cm^–3^. Likewise, **IL3** exhibited average inhibitory effects, with MIC values ranging from 125 to 250 μg·cm^–3^. The observed differences in toxicity can be attributed to variations in alkyl chain length, which impact cell membrane permeability and toxicity towards bacteria and fungi.^48,49^

### Balancing Growth Promotion and Toxicity: Seeking for the Optimal Structure

The search for novel chemical structures with enhanced efficacy is a key aspect of modern agrochemical studies. The development of PGRs that maximize biological activity while minimizing environmental impact is one of the major challenges in sustainable agriculture.^50^ However, one should keep in mind that the introduction of new compounds requires a thorough assessment of their potential toxic effects toward various organisms. Although the increased biological activity of a new substance may seem desirable, its potential environmental harm often makes it unsuitable for commercial use, as shown by the example of neonicotinoids, highly effective insecticides whose use has been restricted in the European Union due to their negative impact on pollinator populations, particularly bees.^51^ Therefore, in this study, the biological activity of the obtained compounds was analyzed, and their environmental safety was assessed by testing the effects on *D. magna* and *A. franciscana* (two recognized bioindicators of aquatic ecosystem health) as well as the soil microorganisms (indicators of soil health). The data in Figure 5A,B show the correlation between compound average plants stimulation efficacy vs their average toxicity toward both aquatic organisms (in each case, the means of the toxicity ranges were used for the calculations) and soil microorganisms (average MIC value for all analyzed bacteria and fungi), respectively. Furthermore, compounds tested did not selectively affect only the development of the plants’ shoot or root system; the observed changes in the development of whole plants were generally consistent. As a consequence, the growth stimulation in Figure 5 was reported as the average value for the shoot and root.

**Figure 5:**
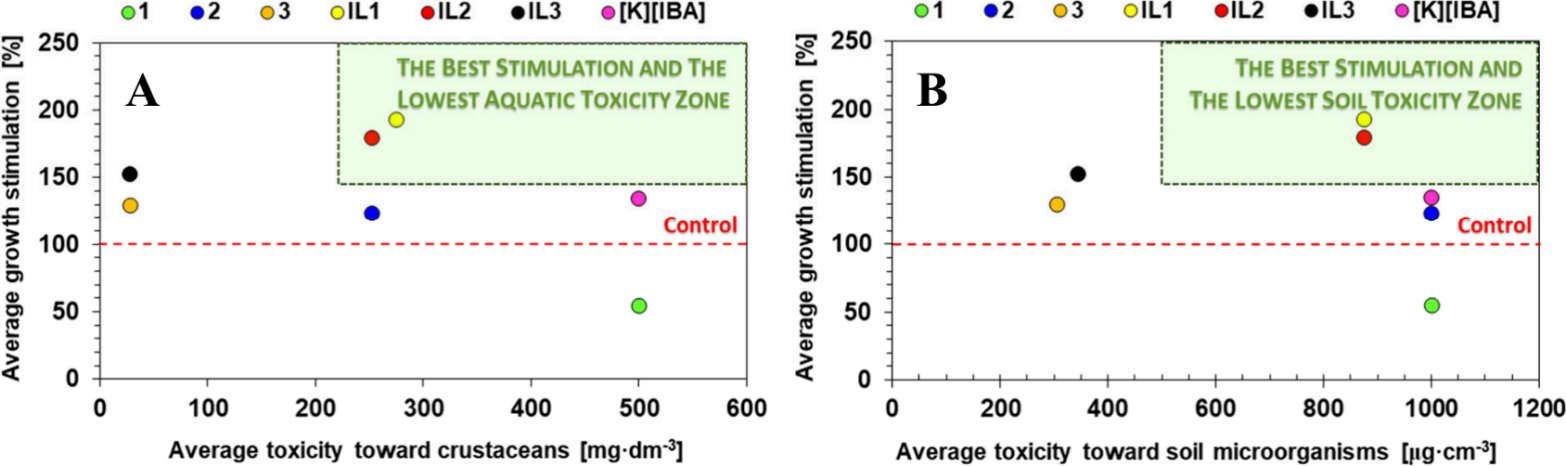
Correlation between average growth stimulation of synthesized compounds with their average toxicity toward crustaceans (*D. magna* and *A. franciscana*) (A) and soil microorganisms (Gram-positive and Gram-negative bacteria and fungi) (B).

The correlation provided reveals that the greatest potential of application is held by **IL1** and **IL2**, which are located in “the best stimulation and the lowest toxicity zone”. Both compounds exhibited increased biological effect compared to **[K]­[IBA]** by approximately 40–60%. Apparently, the increase in growth stimulation led to an undesired increase in toxic effect; their EC_50_ was approximately 50% lower, and MIC was approximately 12% lower. Intriguingly, further alkyl elongation in the IL cation contributed to a substantial increase in the toxic effect with a simultaneous slight increase in growth stimulation of **IL3** compared to **[K]­[IBA]**. This observation unambiguously confirms the importance of optimizing the structure of ionic liquids if they are to be designed to meet the principles of green chemistry.^52^ It should be emphasized that all tested chloride salts (**1**–**3**) caused the weakest biological effect or even a severe disruption of development, which eliminates them as the alternative for commercial plant stimulators based on IBA. However, the combination of IBA with betaine-type cations allowed the achievement of highly promising results while maintaining acceptable levels of toxicity. Therefore, designed **IL1** and **IL2** call for further research as potentially attractive novel agrochemicals for the stimulation of plant development.

## Conclusions

In this study, alkylbetaine and indole-3-butyric acid (IBA) were used for the development of new PGRs with a low environmental impact. Therefore, an efficient and sustainable method of their synthesis was developed. Utilization of known and commercially available reagents allows us to conclude that the synthesis is low cost and demonstrates potential for industrial implementation. In addition, the selected *Green Chemistry* metrics prove the negligible environmental impact of the synthesis of these compounds. Interestingly, the designed **IL1**–**IL3** and chloride salts **1**–**3** showed high purity with only water and potassium chloride as contaminants, which do not pose a risk for agrochemical application. First, assays on white mustard and sorghum showed that solutions of **IL1**–**IL3**, chloride salts **1**–**3** (which are a source of cations), and the potassium salt of IBA had no effect on plant growth when solutions of 5 or 25 ppm of IBA were applied to the soil. An alternative method of applying the active ingredient can instead trigger a diverse effect: if the seeds are soaked in an aqueous solution at a concentration of 25 ppm of IBA for 12 h before sowing, then its stimulating activity toward the plants is observed. From the analysis of early plant development (root and shoot length) at this concentration, it was observed that the compound with the shortest alkyl substituents (**IL1**) was the best candidate as a novel PGR to stimulate growth for monocotyledonous and dicotyledonous plants. These findings pave the way for future studies to determine the effect of these substances at other concentrations applied in the soil and when applied by spraying at later growth stages. Furthermore, the ecotoxicity studies carried out showed that, regardless of the length of the alkyl chain, the products pose a minor negative effect on aquatic life and soil microorganisms. The most promising compound (**IL1**) has similar effects on the aquatic and soil environment as the potassium salt of IBA. This study proves that the design of IL-type PGRs obtained by combining IBA (natural auxins) and alkylbetaine (betaine-based compounds) is an effective tool for creating biologically active compounds that are compatible with sustainability concepts and are capable of protecting seeds from the negative effects of microorganisms on seed development.

## Supplementary Material



## Notes

IR, ^1^H NMR, and ^13^C NMR spectra of synthesized compounds; DSC and TGA thermograms of synthesized compounds; Green Chemistry metrics; effect of the ILs and the chloride salts on shoot and root length (PDF)
