# Supplementary material for: From Plant Material to Environmentally Friendly Plant Growth Stimulators: Betaine-Based Ionic Liquids
Source: ACS Sustain Chem Eng. 2025 Oct 13;13(42):17825–36. doi: 10.1021/acssuschemeng.5c04882 (PMC12570261; doi:10.1021/acssuschemeng.5c04882)
Supplement: Supplementary file 1 [file sc5c04882_si_001.pdf]

Supplementary Information

From Plant Material to Environmentally Friendly Plant Growth Stimulators – Betaine-Based Ionic Liquids

Adriana Olejniczak[a], Michał Niemczak[a], Daniela Gwiazdowska[c], Krzysztof Juś[c], Andrea Mezzetta[b], Lorenzo Guazzelli[b], Damian Krystian Kaczmarek*[,a,b]

[a] Faculty of Chemical Technology, Poznan University of Technology, ul. Berdychowo 4, Poznan 60-965, Poland

[b] Department of Pharmacy, University of Pisa, via Bonanno 6, 56126 Pisa, Italy

[c] Department of Natural Science and Quality Assurance, Poznań University of Economics and Business, al. Niepodległości 10, Poznan 61-875, Poland

* E-mail address: damian.kaczmarek@put.poznan.pl

Number of pages: **26**

Number of figures: **30**

Number of tables: **3**

Table of Contents

1. IR, ^{1}H NMR and ^{13}C NMR spectrum of synthesized compounds **S2–S19**
2. DSC and TGA thermogram of synthesized compounds **S20–S25**
3. *Green Chemistry* metrics for salts **1-3** and ILs **IL1-IL3** **S26**
4. Effect of 25 ppm of IBA aqueous solutions of the ILs **IL1-IL3** and the salts **1-3** on shoot and root length of white mustard and sorghum **S26**

Figure S.1. ^{1}H NMR spectrum of octyldimethylglycinium hydrochloride ***(1)***

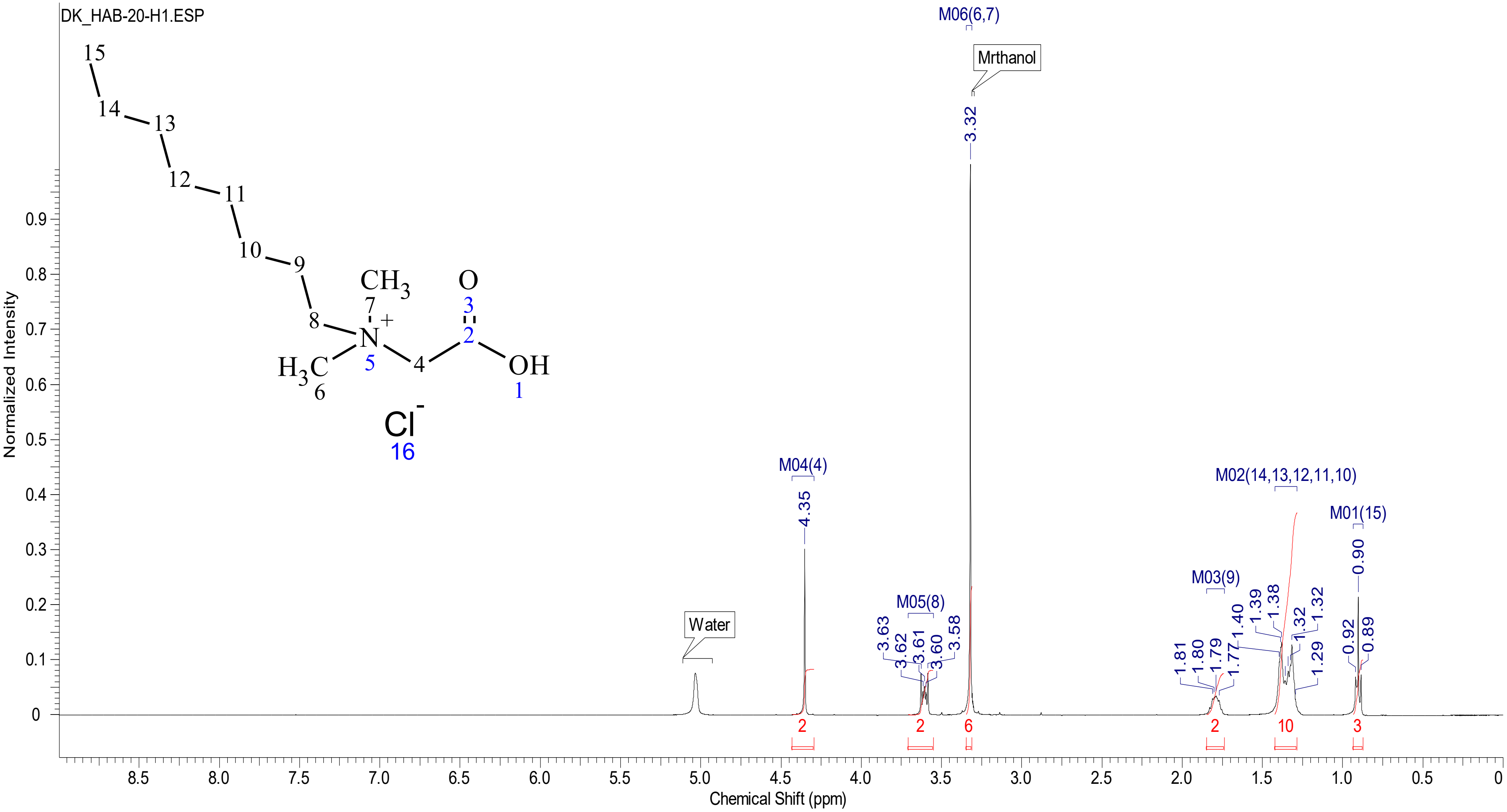

^{1}H NMR (400 MHz, METHANOL-d_4) δ [ppm] = 4.35 (2H, s, H-4), 3.55 - 3.71 (2H, m, H-8), 3.32 (6H, s, H-6, 7), 1.74 - 1.85 (2H, m, H-9), 1.28 - 1.42 (10H, m, H-14, 13, 12, 11, 10), 0.87 - 0.94 (3H, m, H-15)

Figure S.2. ^{13}C NMR spectrum of octyldimethylglycinium hydrochloride ***(1)***

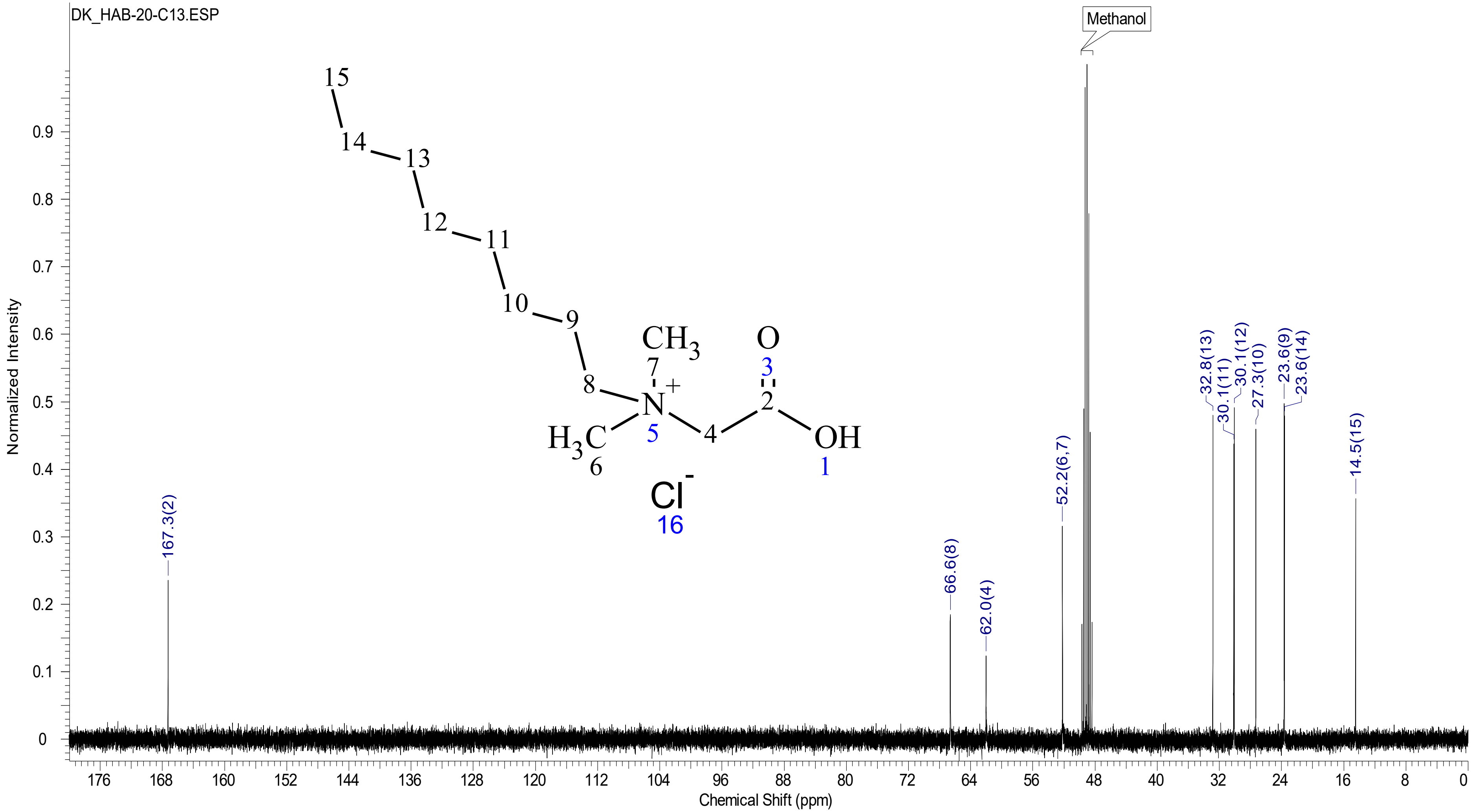

^{13}C NMR (100 MHz, METHANOL-*d₄*) δ [ppm] = 14.5; 23.6 (2C); 27.3; 30.1 (2C); 32.8; 52.2 (2C); 62.0; 66.6; 167.3.

Figure S.3. IR spectrum of octyldimethylglycinium hydrochloride ***(1)***

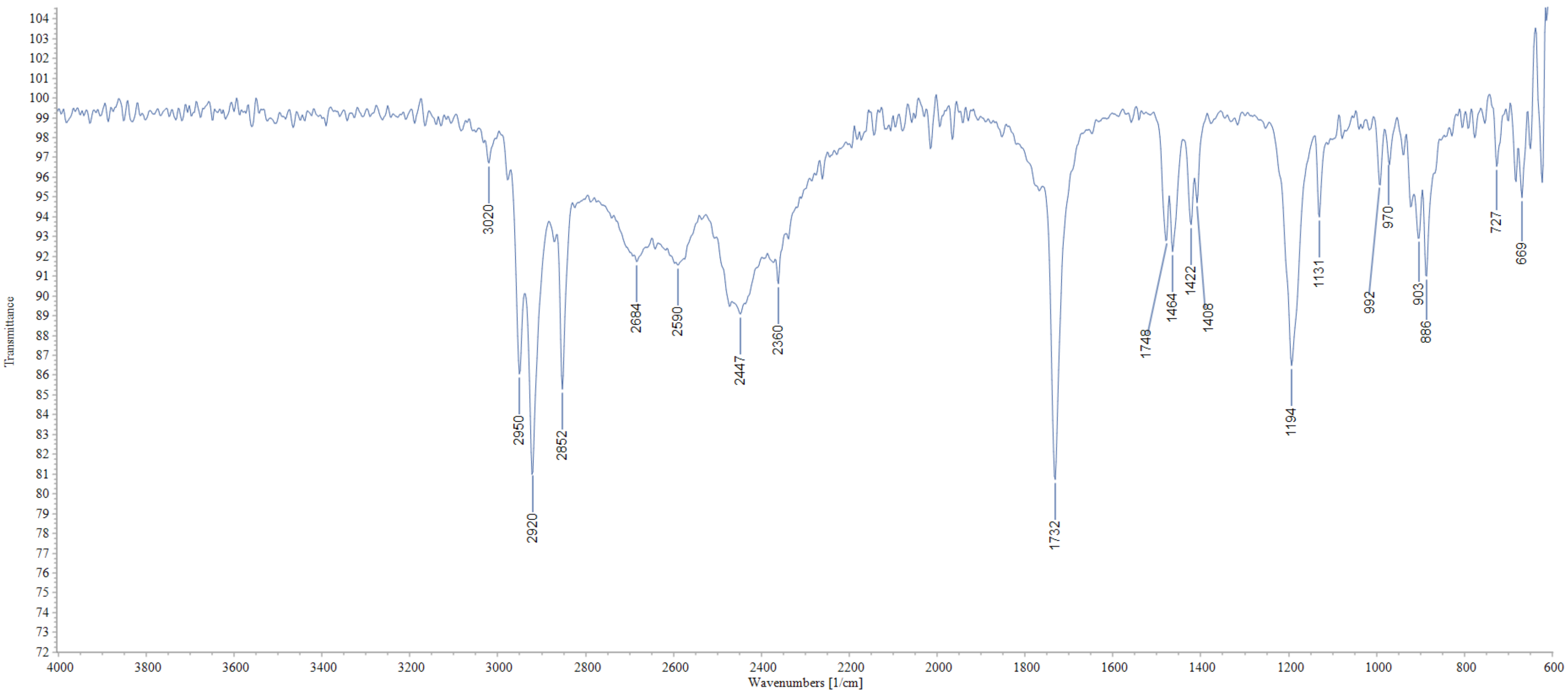

Figure S.4. ^{1}H NMR spectrum of decyldimethylglycinium hydrochloride ***(2)***

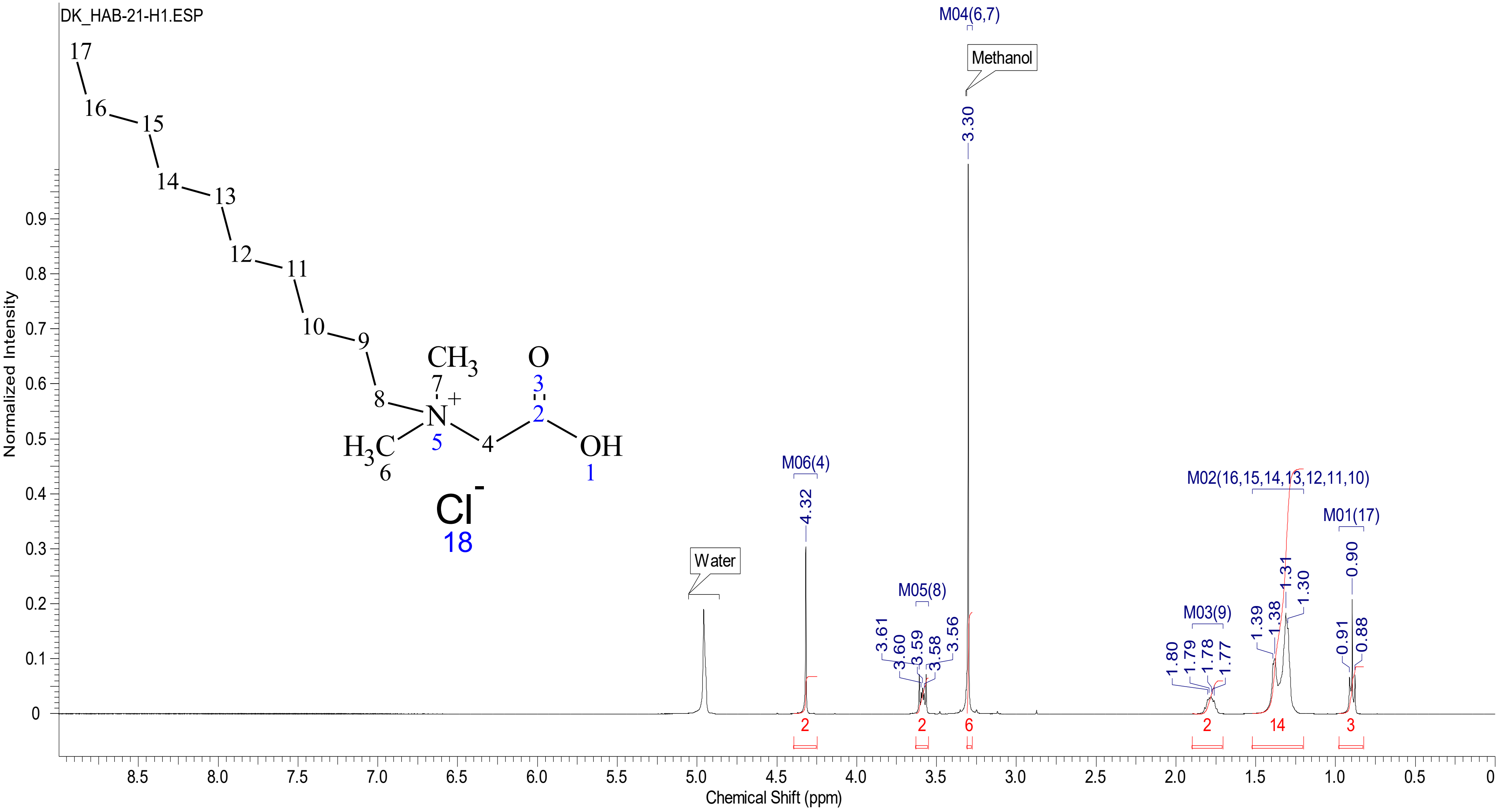

^{1}H NMR (400 MHz, METHANOL-*d_4*) δ [ppm] = 4.32 (3H, s, H-4), 3.55 - 3.63 (2H, m, H-8), 3.30 (6H, s, H-6, 7), 1.70 - 1.90 (2H, m, H-9), 1.20 - 1.52 (18H, m, H-16, 15, 14, 13, 12, 11, 10), 0.82 - 0.98 (3H, m, H-17)

Figure S.5. ^{13}C NMR spectrum of decyldimethylglycinium hydrochloride ***(2)***

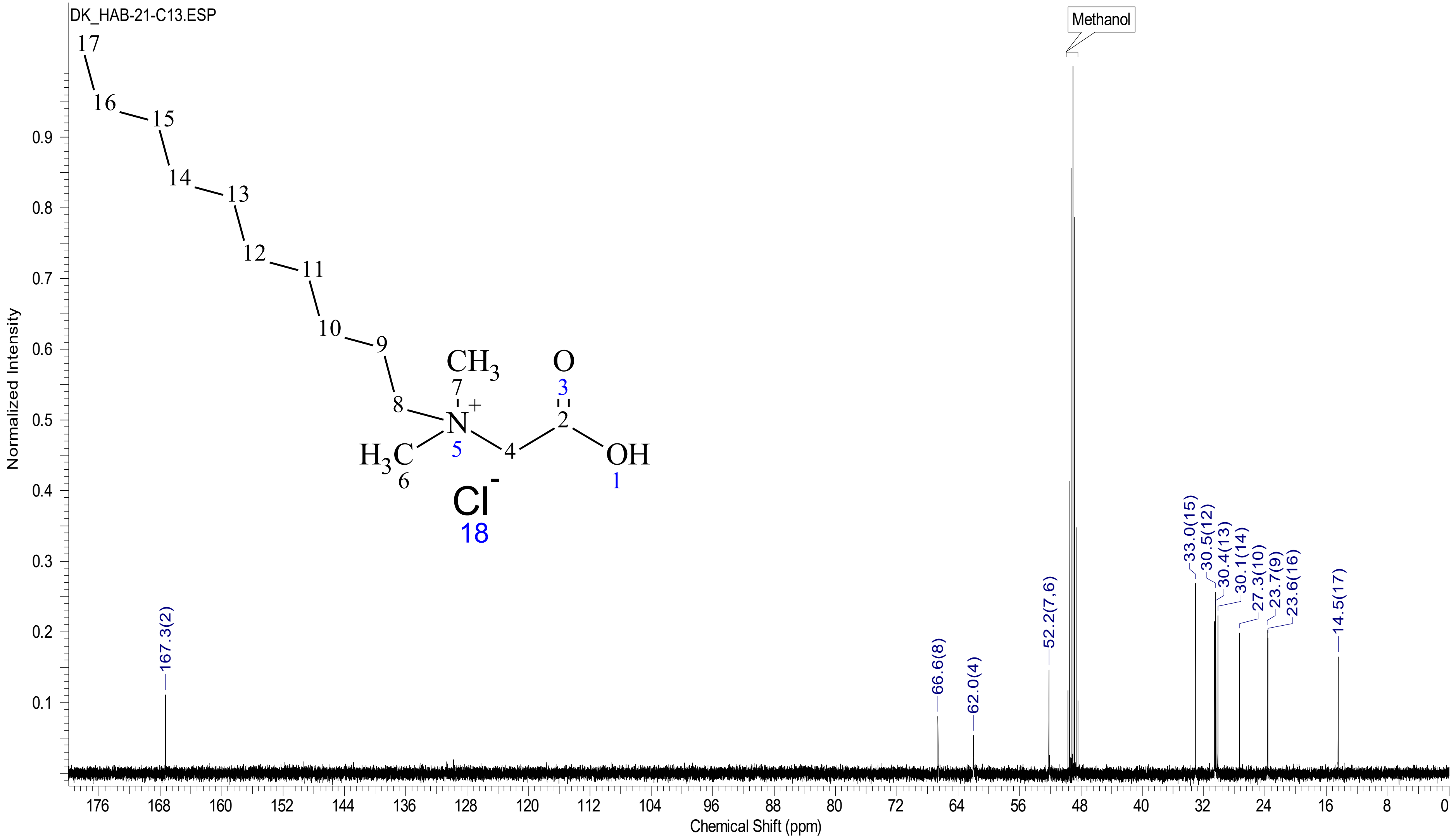

^{13}C NMR (100 MHz, METHANOL-*d*$_4$) δ [ppm] = 14.5; 23.6; 23.7; 27.3; 30.1; 30.4; 30.5; 30.6; 33.0; 52.2 (2C); 62.0; 66.6; 167.3.

Figure S.6. IR spectrum of decyldimethylglycinium hydrochloride ***(2)***

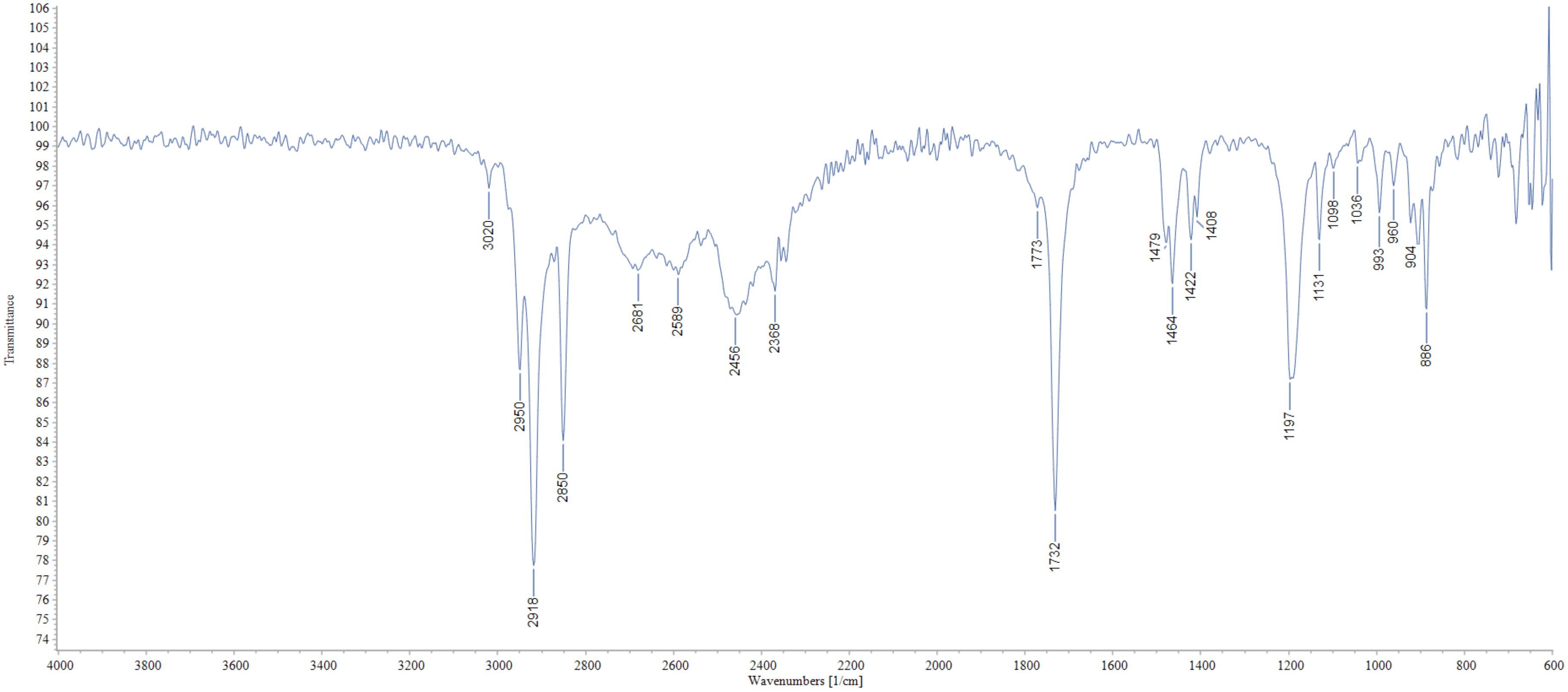

Figure S.7. ^{1}H NMR spectrum of dodecyldimethylglycinium hydrochloride ***(3)***

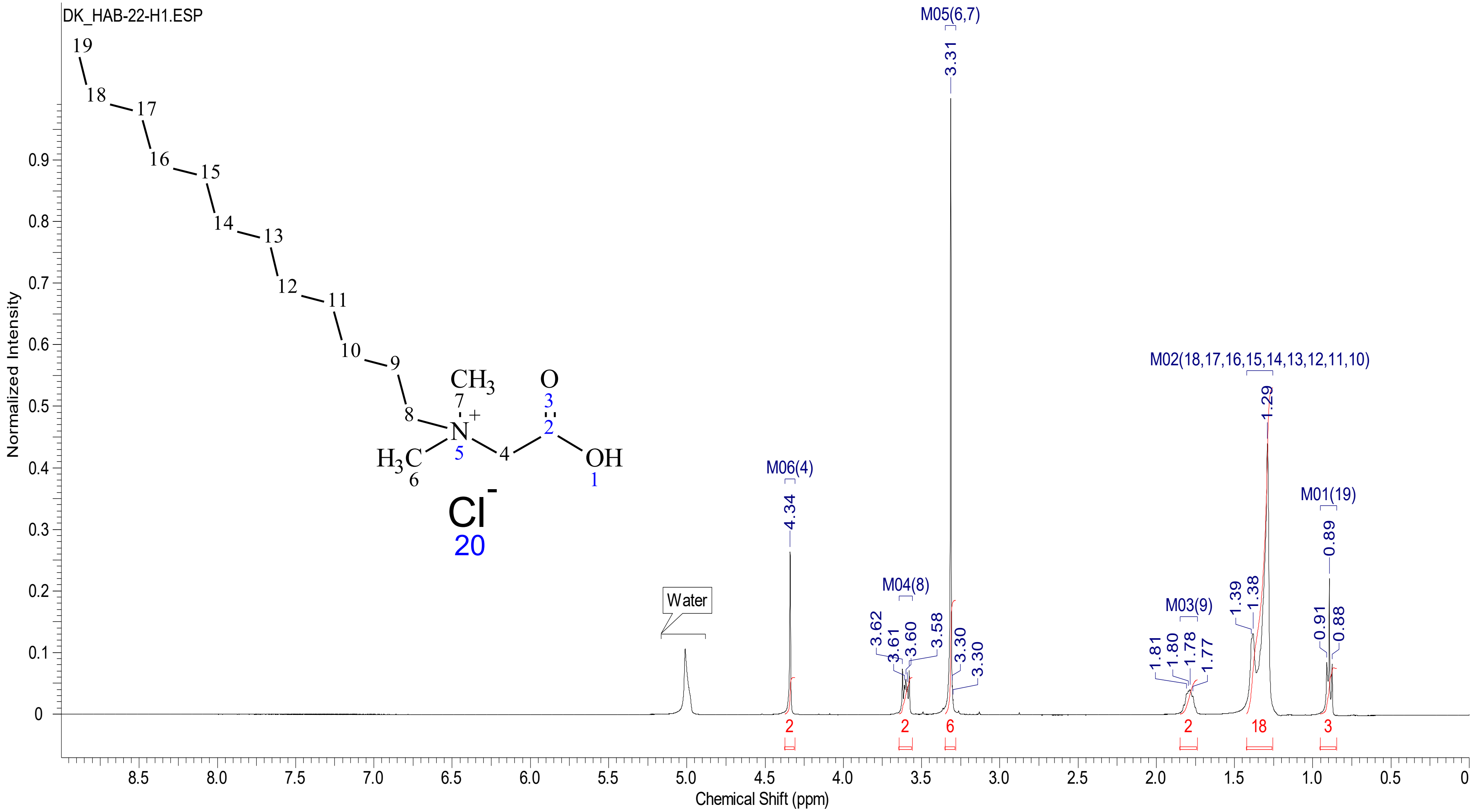

^{1}H NMR (400 MHz, METHANOL-*d₄*) δ [ppm] = 4.34 (2H, s, H-4), 3.56 - 3.64 (2H, m, H-8), 3.28 - 3.35 (6H, m, H-6, 7), 1.74 - 1.85 (2H, m, H-9), 1.26 - 1.42 (18H, m, H-18, 17, 16, 15, 14, 13, 12, 11, 10), 0.85 - 0.95 (3H, m, H-19)

Figure S.8. ^{13}C NMR spectrum of dodecyldimethylglycinium hydrochloride ***(3)***

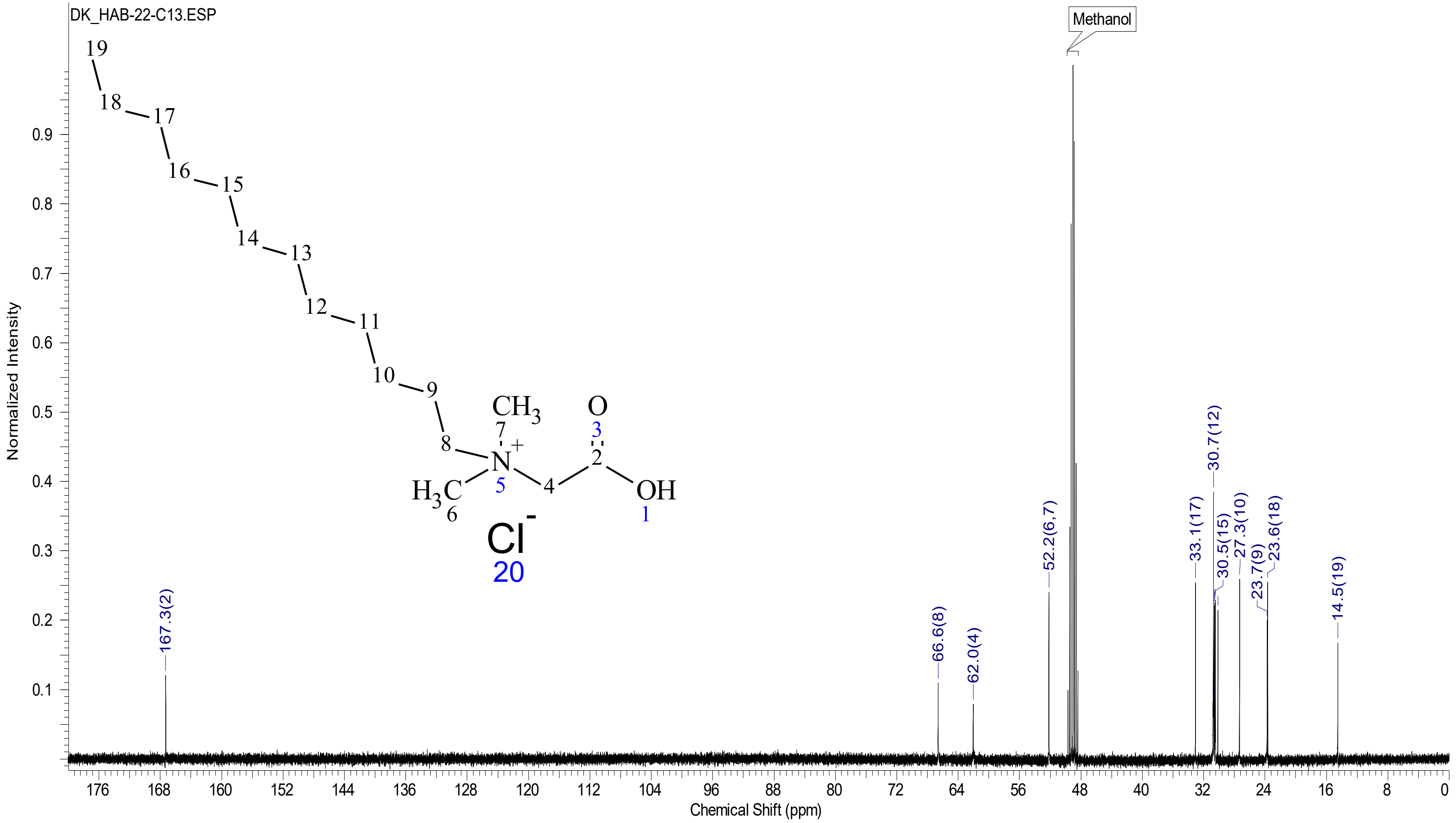

^{13}C NMR (100 MHz, METHANOL-*d*$_4$) δ [ppm] = 14.5; 23.6; 23.7; 27.3; 30.1; 30.5 (2C); 30.6; 30.7; 30.8; 33.1; 52.2 (2C); 62.0; 66.6; 167.3.

Figure S.9. IR spectrum of dodecyldimethylglycinium hydrochloride *(3)*

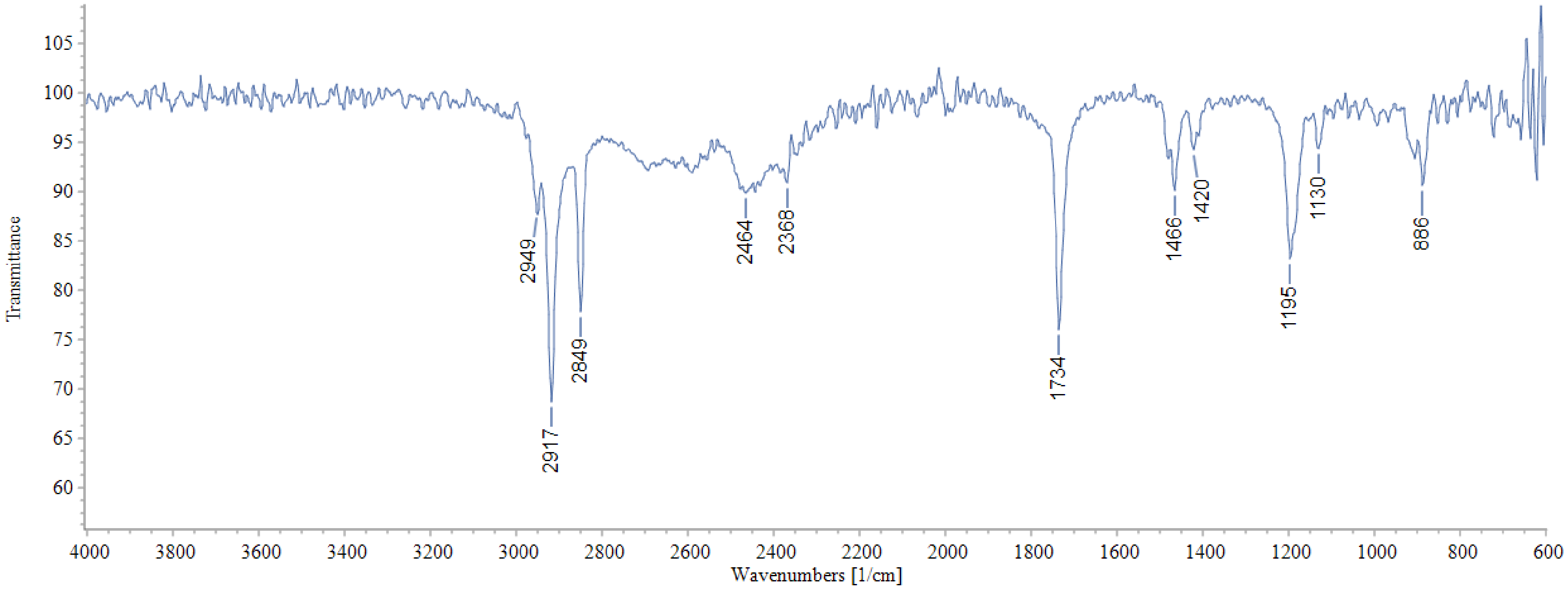

Figure S.10. ^{1}H NMR spectrum of octyldimethylglycinium indole-3-butyrate ***(IL1)***

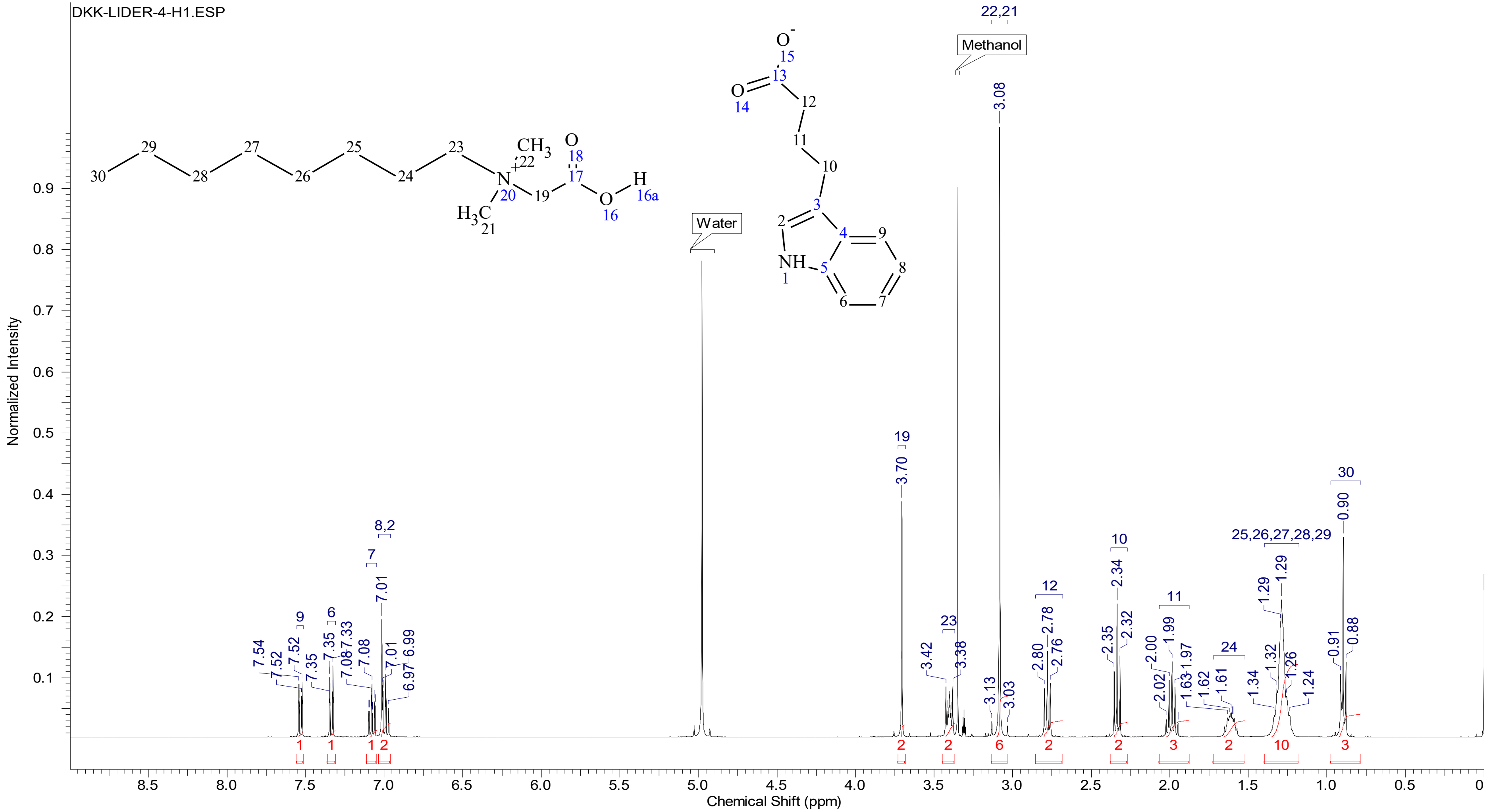

^{1}H NMR (400 MHz, METHANOL-*d₄*) δ [ppm] = 7.53 (1H, dt, J = 7.9 Hz, J = 0.9 Hz, H-9), 7.34 (1H, dt, J = 8.1 Hz, J = 0.9 Hz, H-6), 7.05 - 7.11 (1H, m, H-7), 6.96 - 7.03 (2H, m, H-8, 2), 3.70 (2H, s, H-19), 3.37 - 3.45 (2H, m, H-23), 3.03 - 3.13 (5H, m, H-22, 21), 2.68 - 2.85 (2H, m, H-12), 2.33 (2H, t, J = 7.4 Hz, H-10), 1.99 (2H, quin, J = 7.5 Hz, H-11), 1.52 - 1.72 (2H, m, H-24), 1.18 - 1.40 (10H, m, H-25, 26, 27, 28, 29), 0.78 - 0.98 (3H, m, H-30)

Figure S.11. ^{13}C NMR spectrum of octyldimethylglycinium indole-3-butyrate ***(IL1)***

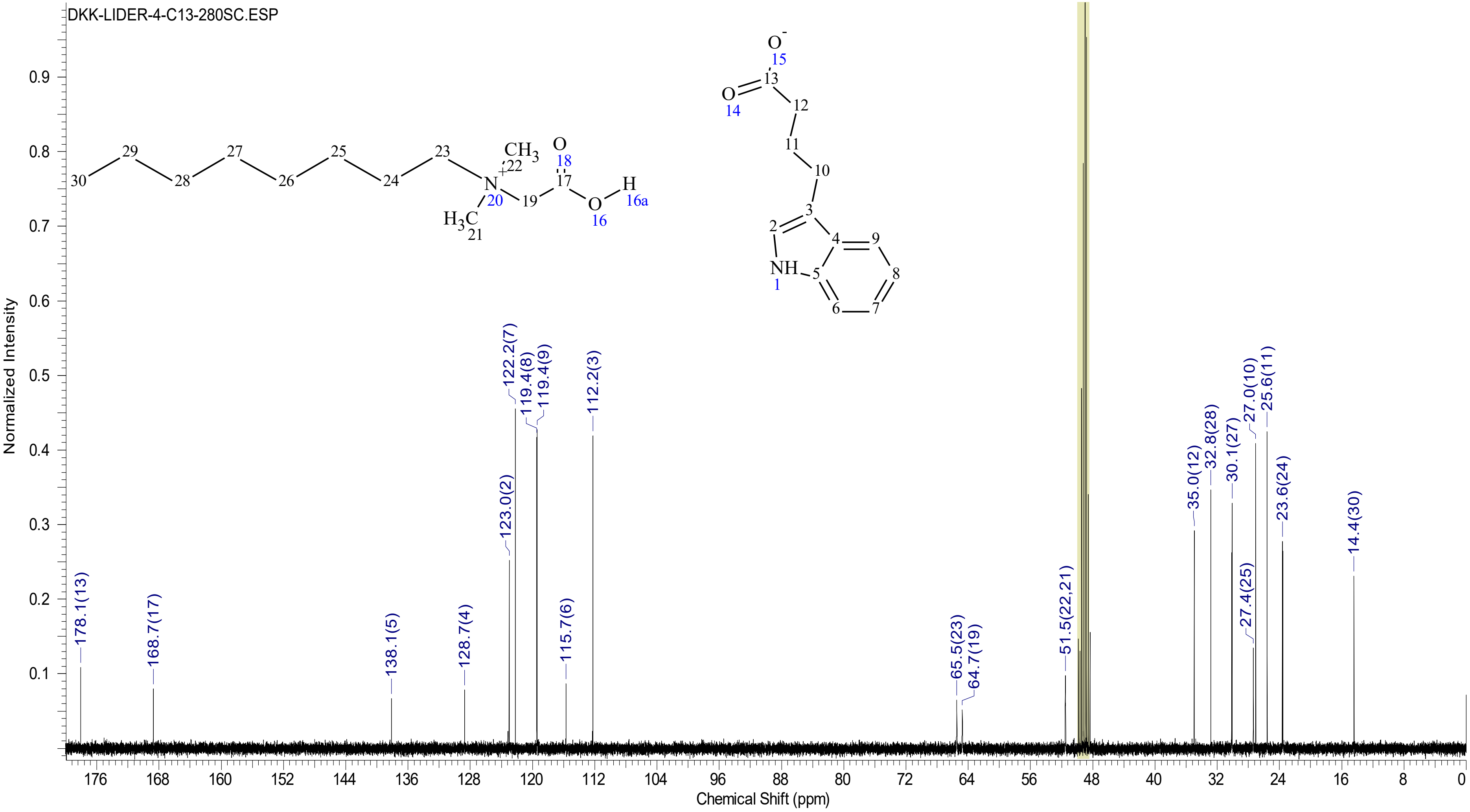

^{13}C NMR (100 MHz, METHANOL-*d₄*) δ [ppm] = 14.4; 23.6 (2C); 25.6; 27.0; 27.4; 30.1 (2C); 32.8; 35.0; 51.5 (2C); 64.7; 65.5; 112.2; 115.7; 119.4 (2C); 122.2; 123.0; 128.7; 138.1; 168.7; 178.1.

Figure S.12. IR spectrum of octyldimethylglycinium indole-3-butyrate ***(IL1)***

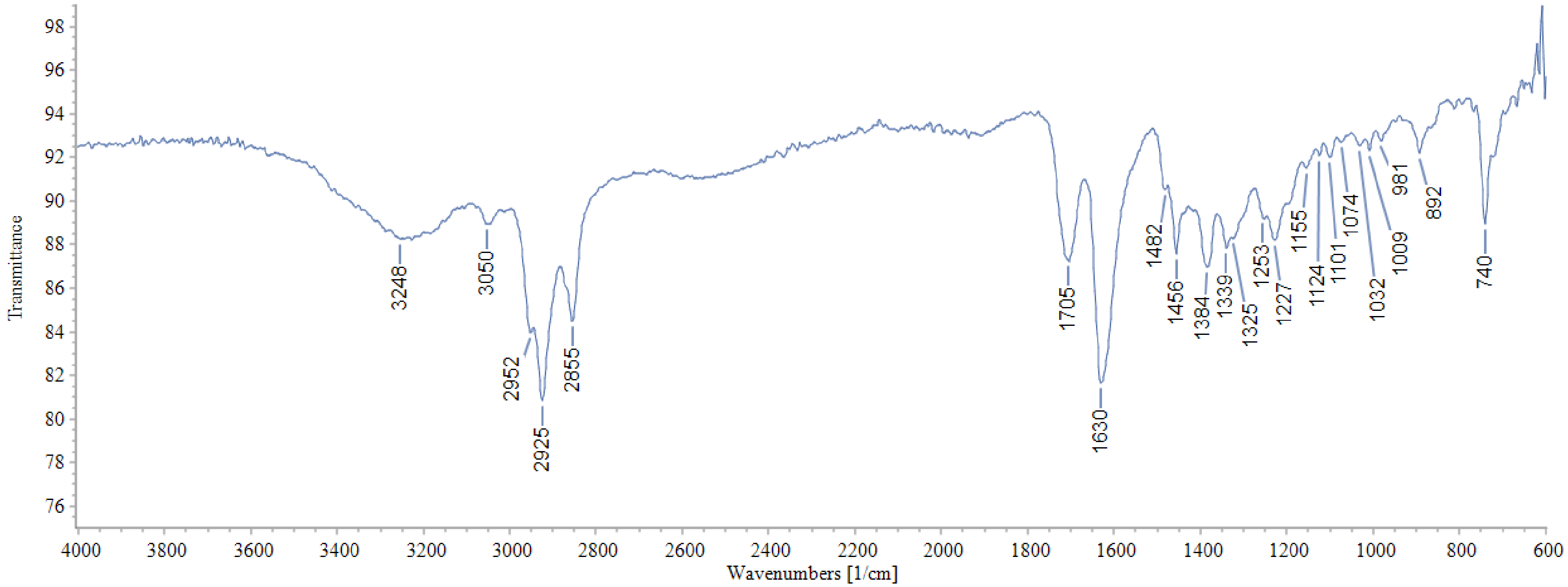

Figure S.13. ^{1}H NMR spectrum of decyldimethylglycinium indole-3-butyrate ***(IL2)***

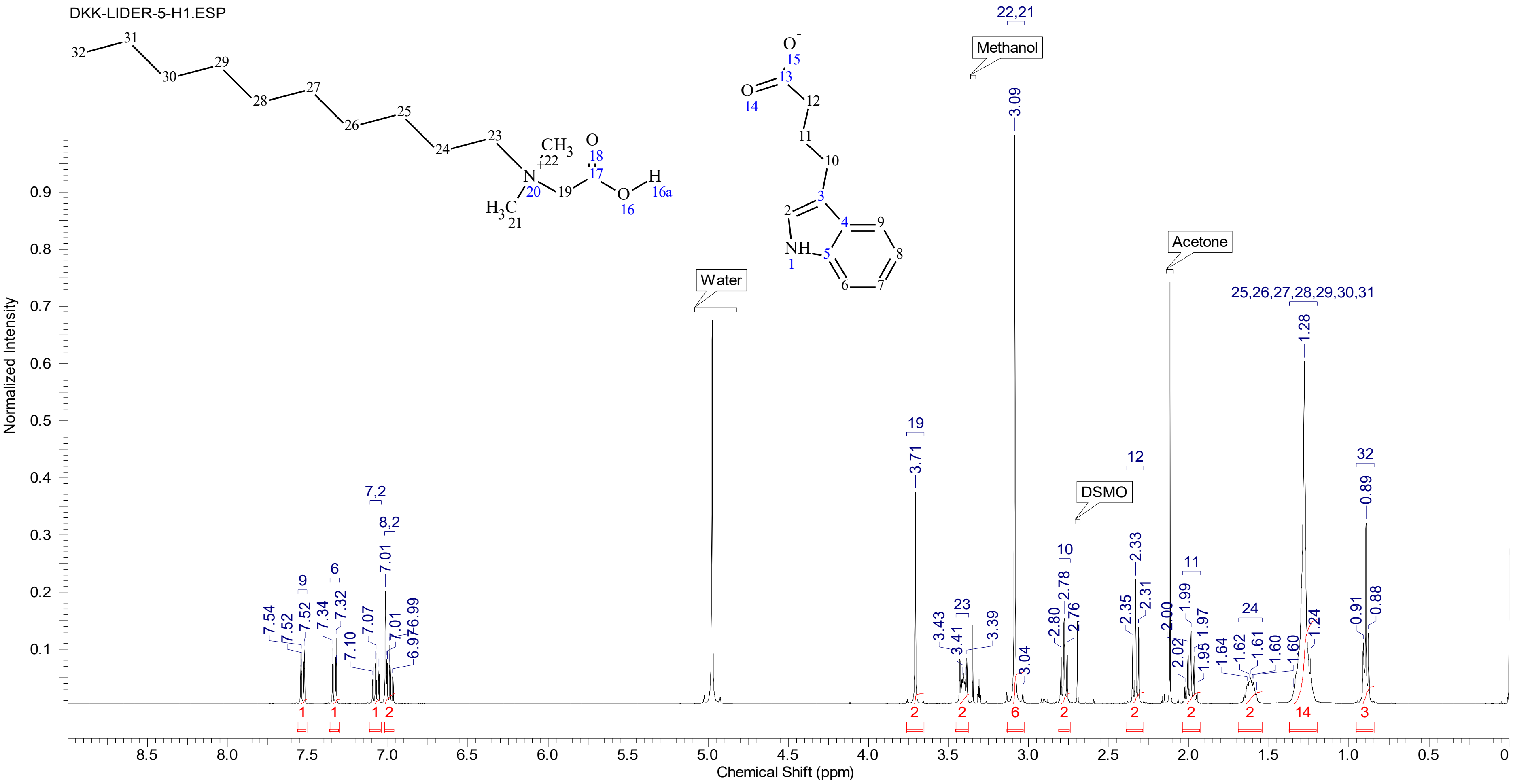

^{1}H NMR (400 MHz, METHANOL-*d₄*) δ [ppm] = 7.53 (1H, dt, J = 7.9 Hz, J = 0.9 Hz, H-9), 7.30 - 7.36 (2H, m, H-6), 7.07 (2H, ddd, J = 8.1 Hz, J = 7.0 Hz, J = 1.2 Hz, H-7, 2), 6.96 - 7.02 (4H, m, H-8, 2), 3.71 (4H, s, H-19), 3.37 - 3.45 (4H, m, H-23), 3.03 - 3.13 (8H, m, H-22, 21), 2.78 (3H, t, J = 7.5 Hz, H-10), 2.33 (3H, t, J = 7.4 Hz, H-12), 1.99 (2H, quin, J = 7.5 Hz, H-11), 1.54 - 1.69 (2H, m, H-24), 1.20 - 1.37 (14H, m, H-25, 26, 27, 28, 29, 30, 31), 0.84 - 0.95 (3H, m, H-32)

Figure S.14. ^{13}C NMR spectrum of decyldimethylglycinium indole-3-butyrate ***(IL2)***

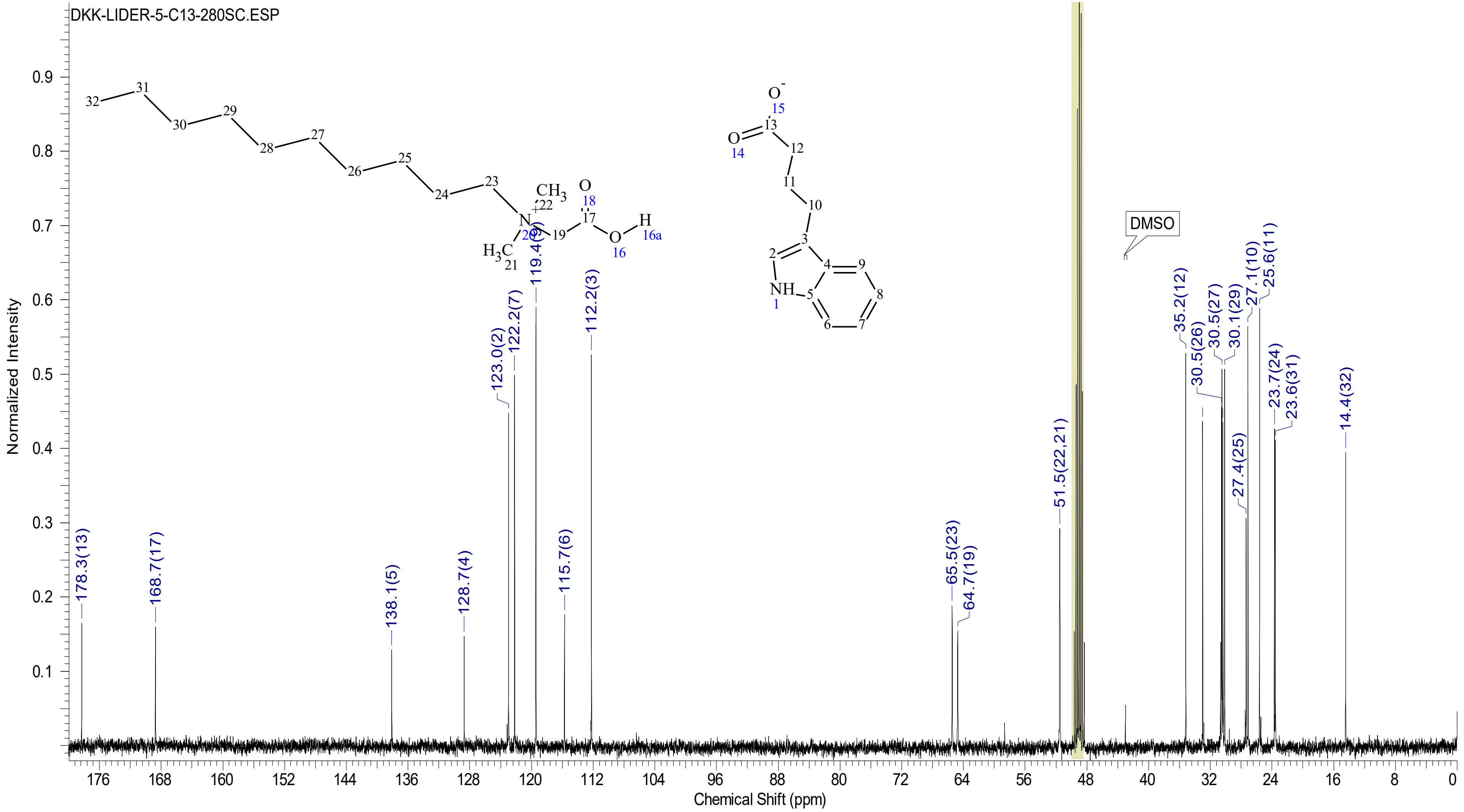

^{13}C NMR (100 MHz, METHANOL-*d₄*) δ [ppm] = 14.4; 23.6; 23.7; 25.6; 27.1; 27.4; 30.1; 30.3; 30.5 (2C); 33.0; 35.2; 51.5 (2C); 64.7; 65.5; 112.2; 115.7; 119.4 (2C); 122.2; 123.0; 128.7; 138.1; 168.7; 178.3.

Figure S.15. IR spectrum of decyldimethylglycinium indole-3-butyrate ***(IL2)***

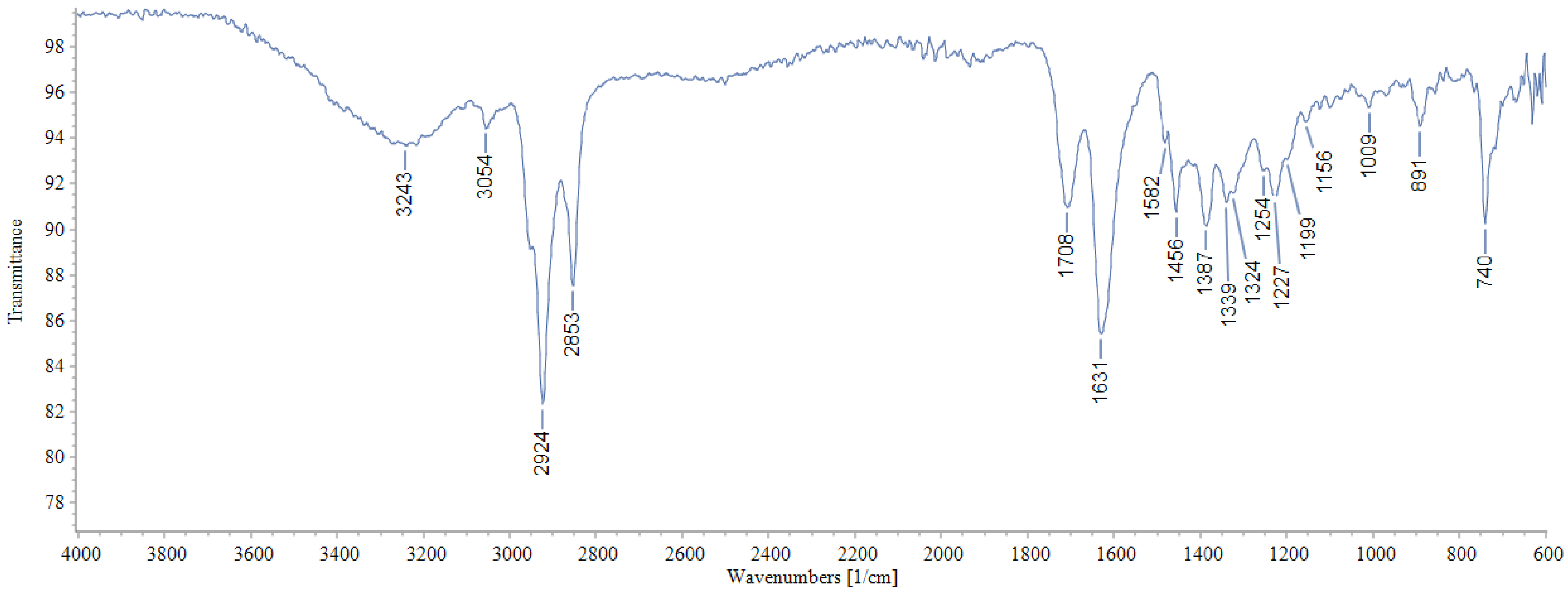

Figure S.16. ^{1}H NMR spectrum of dodecyldimethylglycinium indole-3-butyrate ***(IL3)***

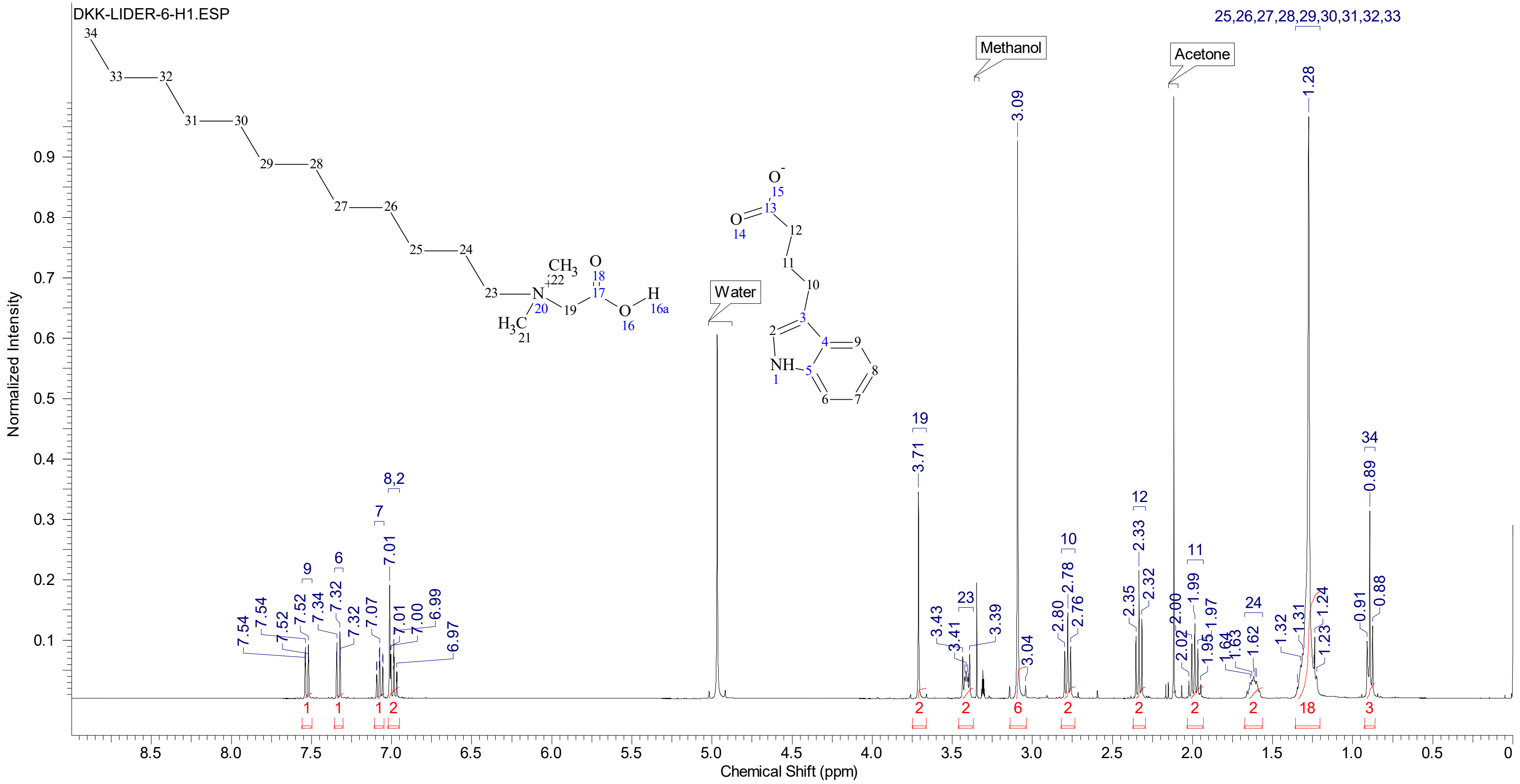

^{1}H NMR (400 MHz, METHANOL-*d₄*) δ [ppm] = 7.53 (1H, dt, J = 7.9 Hz, J = 0.9 Hz, H-9), 7.33 (1H, dt, J = 8.1 Hz, J = 0.8 Hz, H-6), 7.07 (1H, ddd, J = 8.1 Hz, J = 7.0 Hz, J = 1.2 Hz, H-7), 6.95 - 7.02 (2H, m, H-8, 2), 3.71 (2H, s, H-19), 3.37 - 3.46 (2H, m, H-23), 3.04 - 3.14 (6H, m, H-22, 21), 2.78 (2H, t, J = 7.4 Hz, H-10), 2.33 (2H, t, J = 7.4 Hz, H-12), 1.99 (2H, quin, J = 7.4 Hz, H-11), 1.62 (2H, dt, J = 8.0 Hz, J = 3.9 Hz, H-24), 1.20 - 1.36 (18H, m, H-25, 26, 27, 28, 29, 30, 31, 32, 33), 0.86 - 0.92 (3H, m, H-34)

Figure S.17. IR spectrum of dodecyldimethylglycinium indole-3-butyrate ***(IL3)***

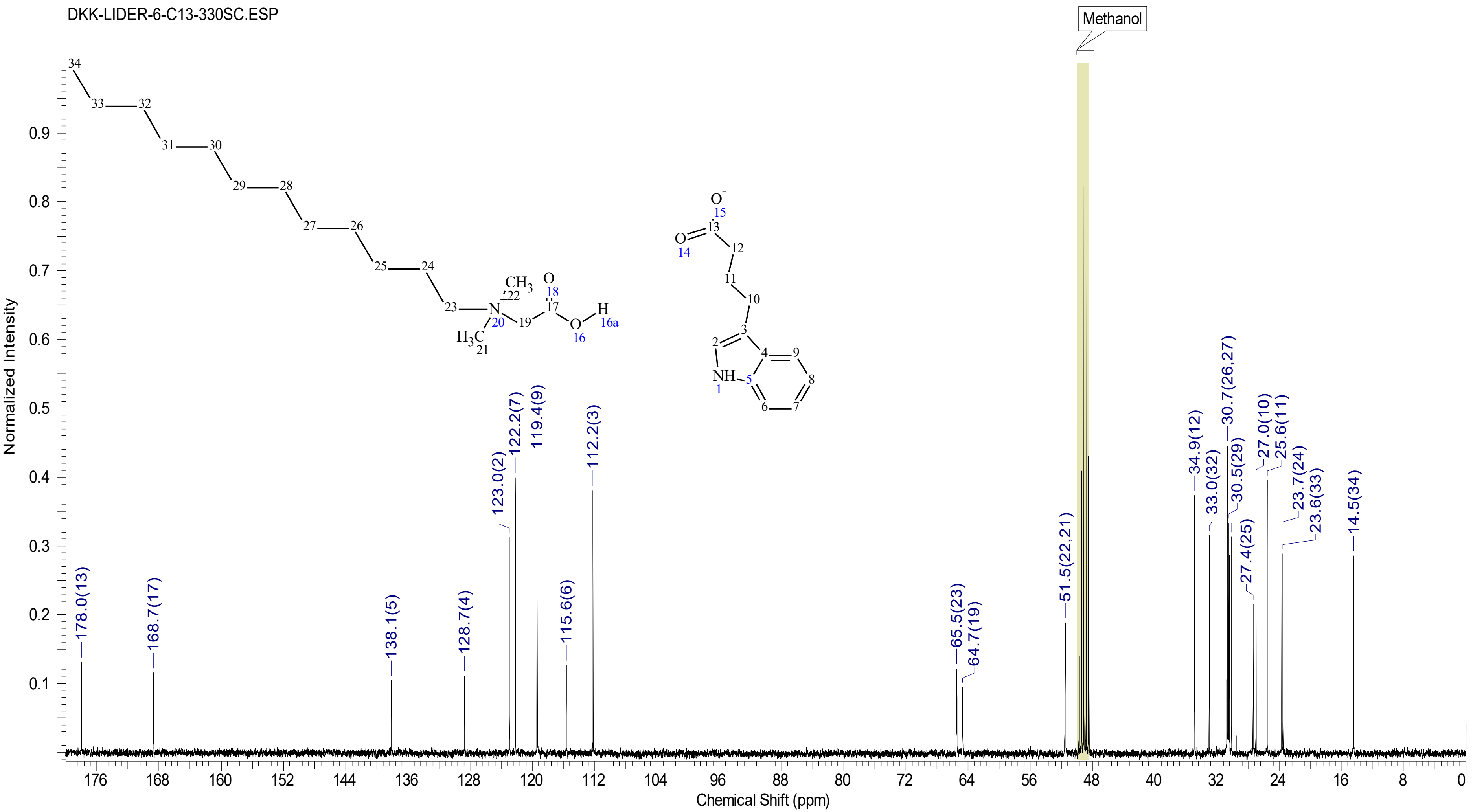

^{13}**C NMR (100 MHz, METHANOL-*d₄*) δ [ppm]** = 14.5; 23.6; 23.7; 25.6; 27.0; 27.4; 30.1; 30.4; 30.5; 30.6; 30.7; 33.0; 34.9; 51.5 (2C); 64.7; 65.5; 112.2; 115.6; 119.4 (2C); 122.2; 123.0; 128.7; 138.1; 168.7; 178.0.

Figure S.18. ^{13}C NMR spectrum of dodecyldimethylglycinium indole-3-butyrate ***(IL3)***

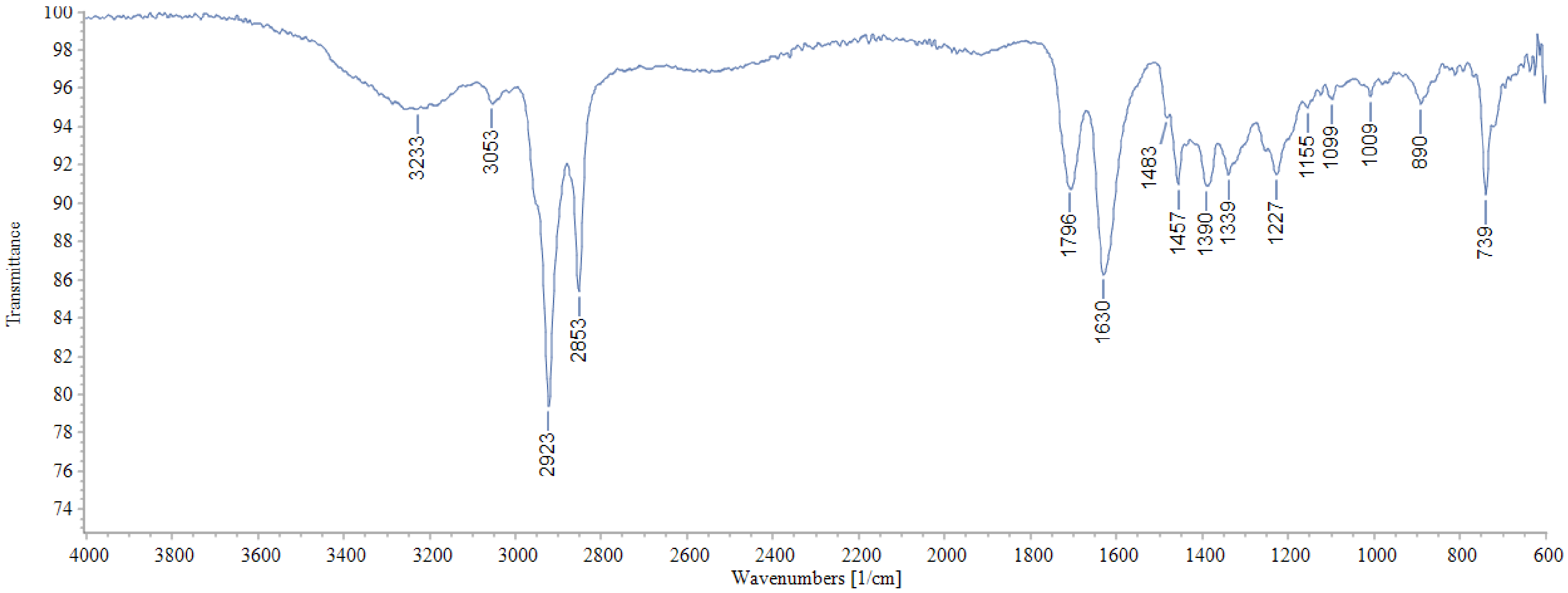

Figure S.19. DSC thermogram of octyldimethylglycinium hydrochloride ***(1)***

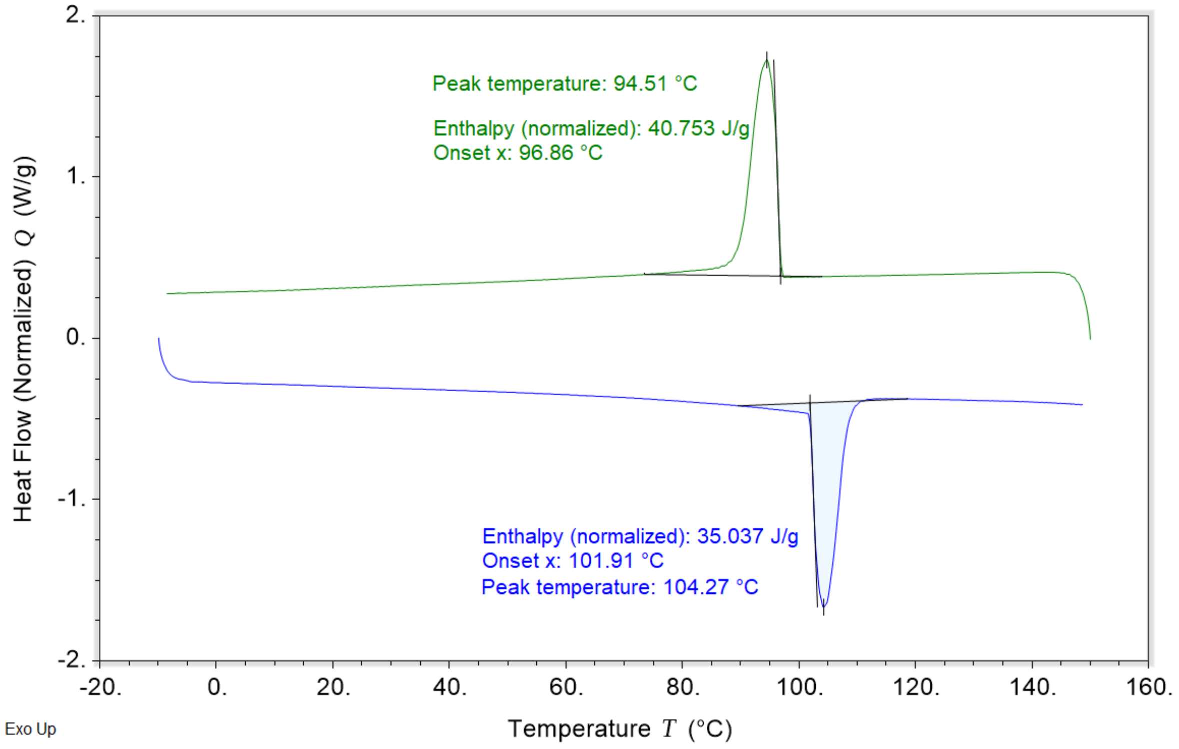

Figure S.20. DSC thermogram of decyldimethylglycinium hydrochloride ***(2)***

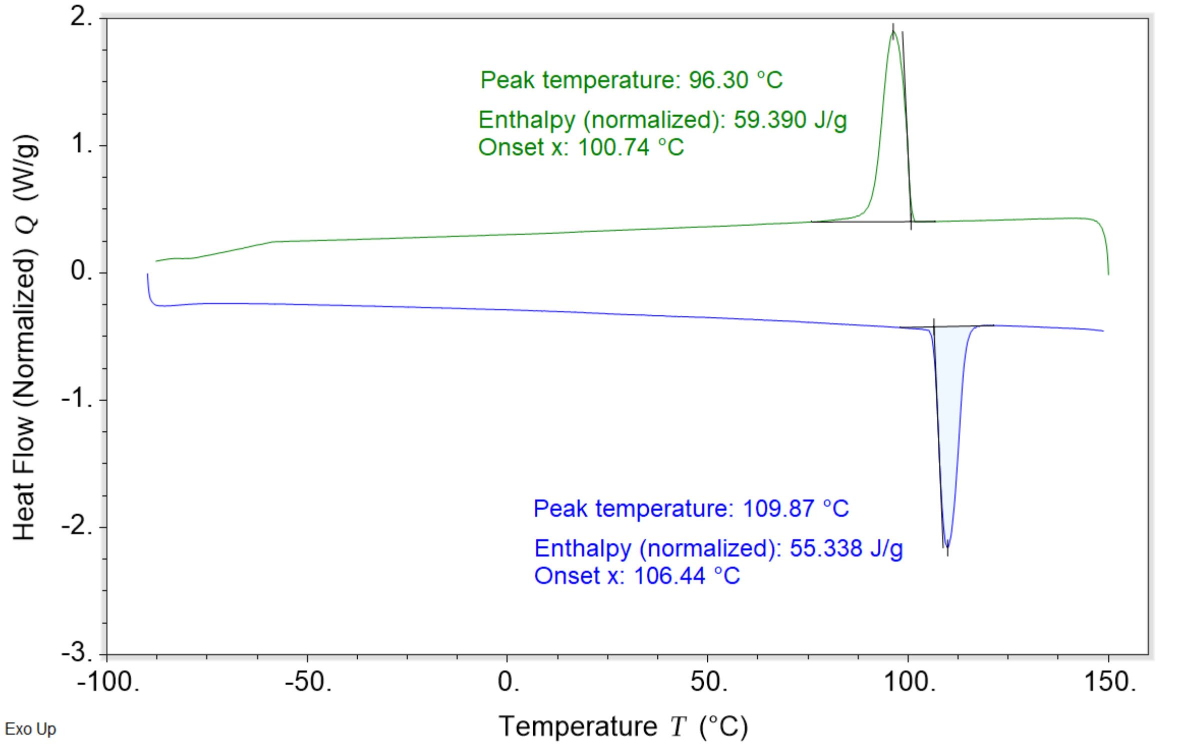

Figure S.21. DSC thermogram of dodecyldimethylglycinium hydrochloride ***(3)***

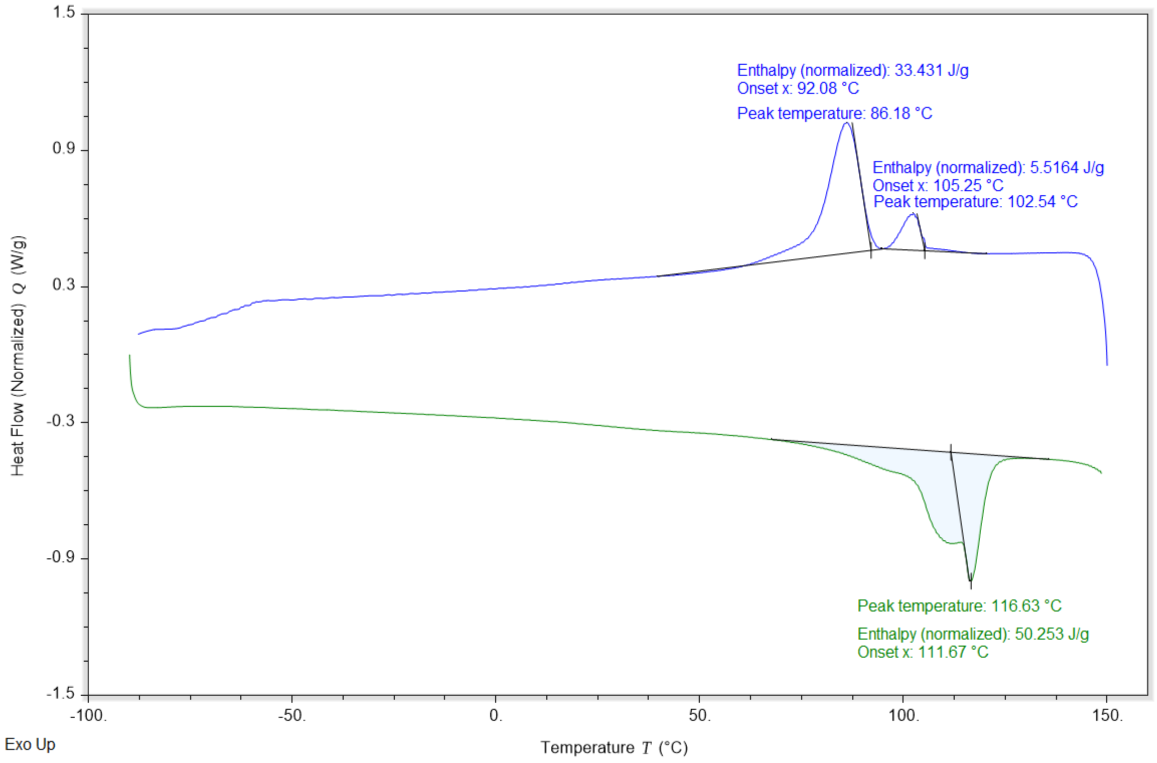

Figure S.22. DSC thermogram of octyldimethylglycinium indole-3-butyrate ***(IL1)***

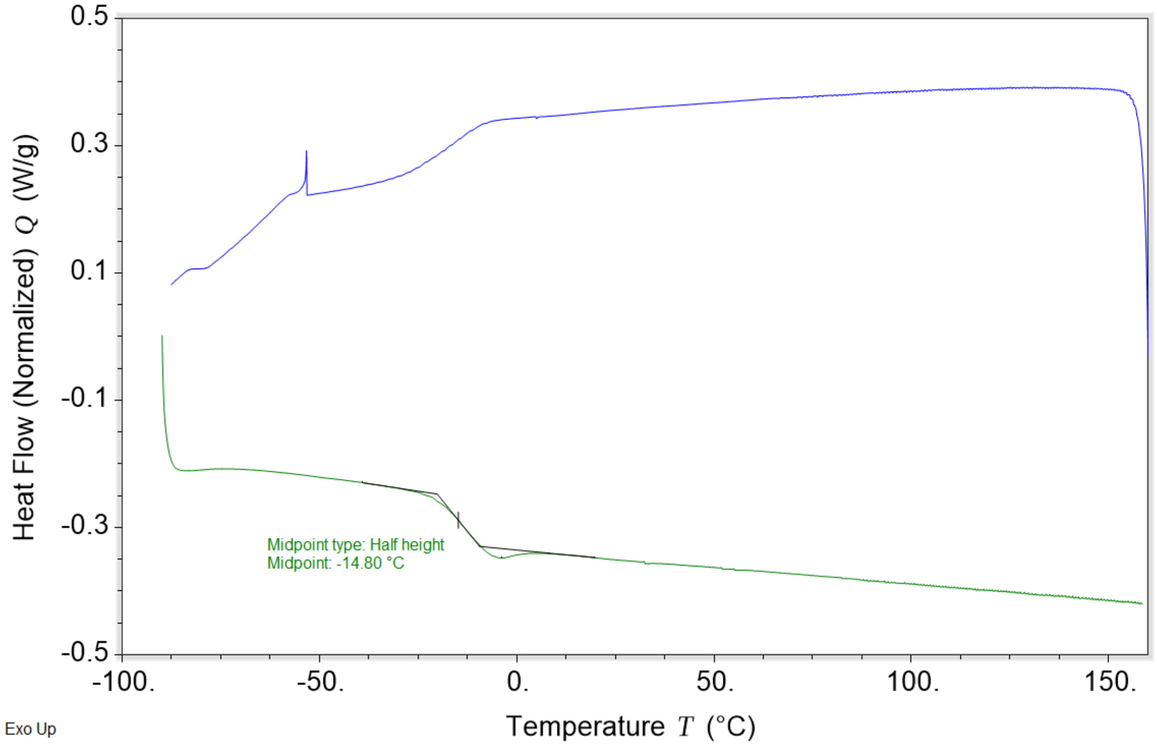

Figure S.23. DSC thermogram of decyldimethylglycinium indole-3-butyrate ***(IL2)***

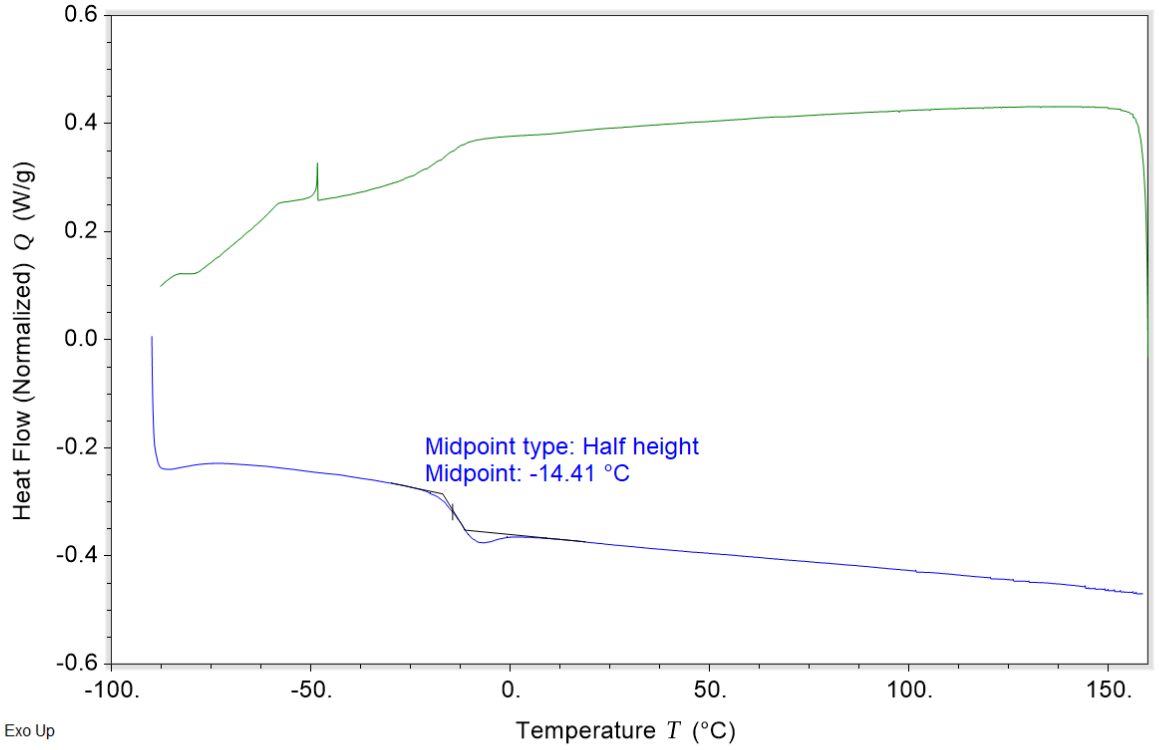

Figure S.24. DSC thermogram of dodecyldimethylglycinium indole-3-butyrate ***(IL3)***

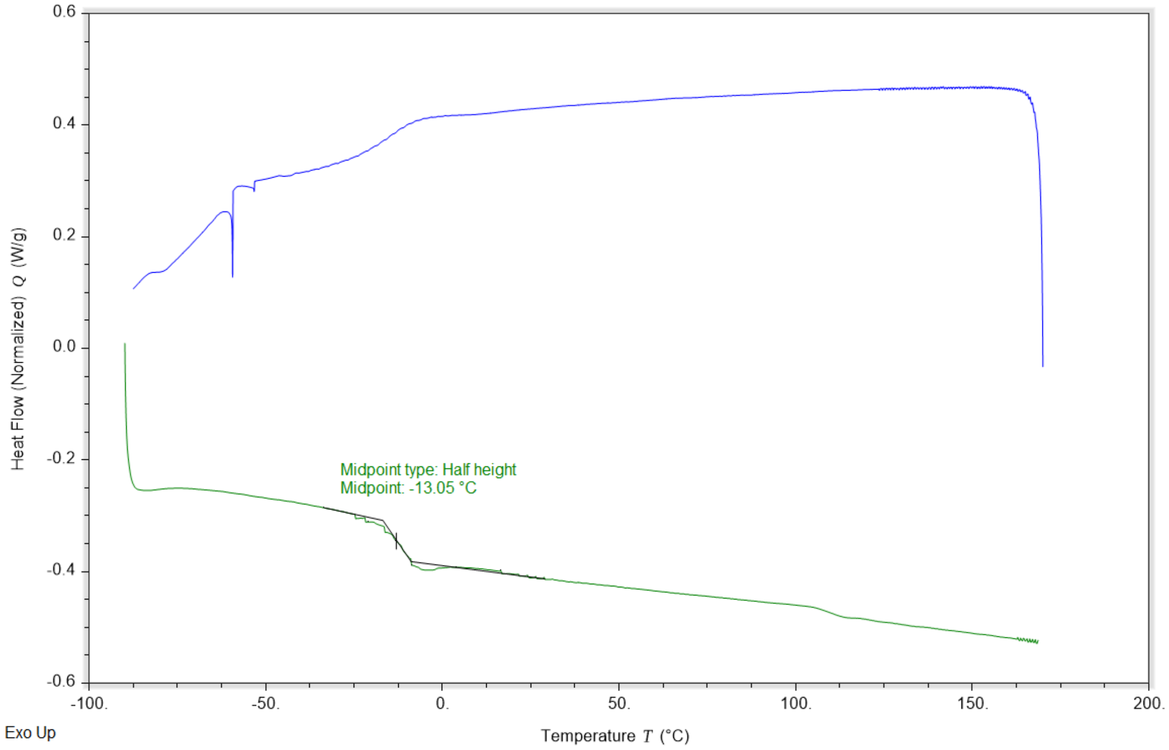

Figure S.25. TGA thermogram of octyldimethylglycinium hydrochloride ***(1)***

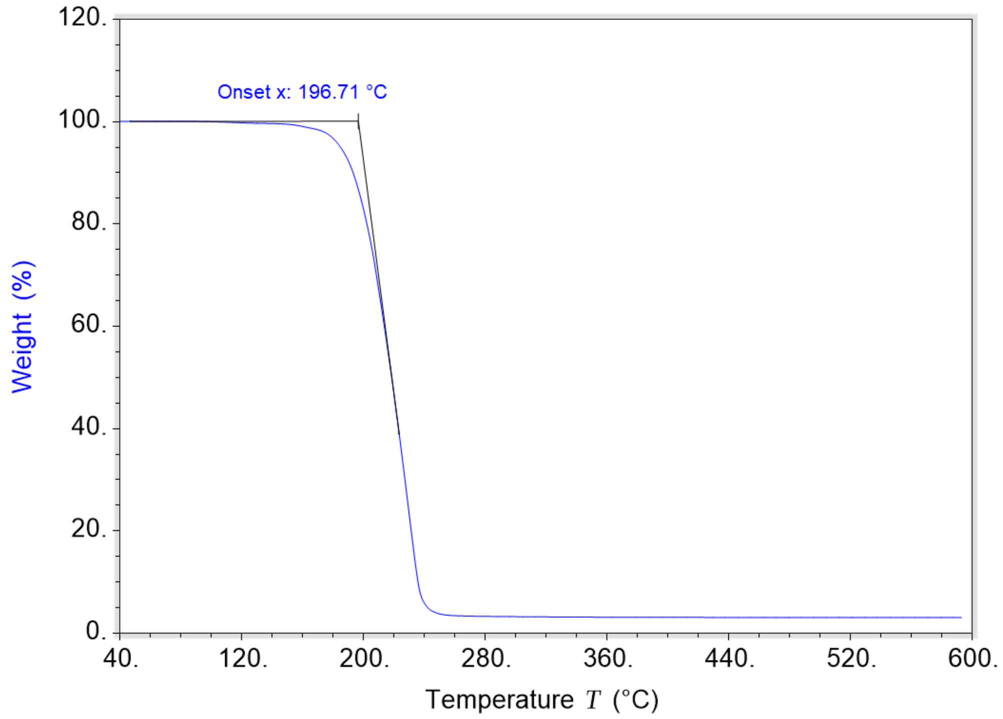

Figure S.26. TGA thermogram of decyldimethylglycinium hydrochloride ***(2)***

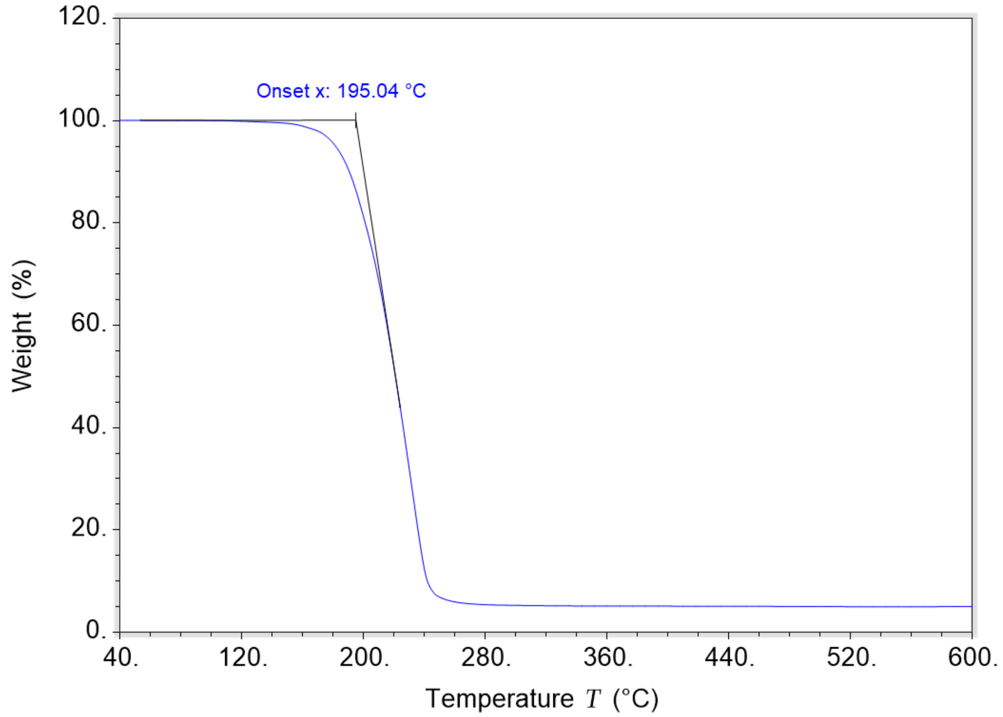

Figure S.27. TGA thermogram of dodecyldimethylglycinium hydrochloride ***(3)***

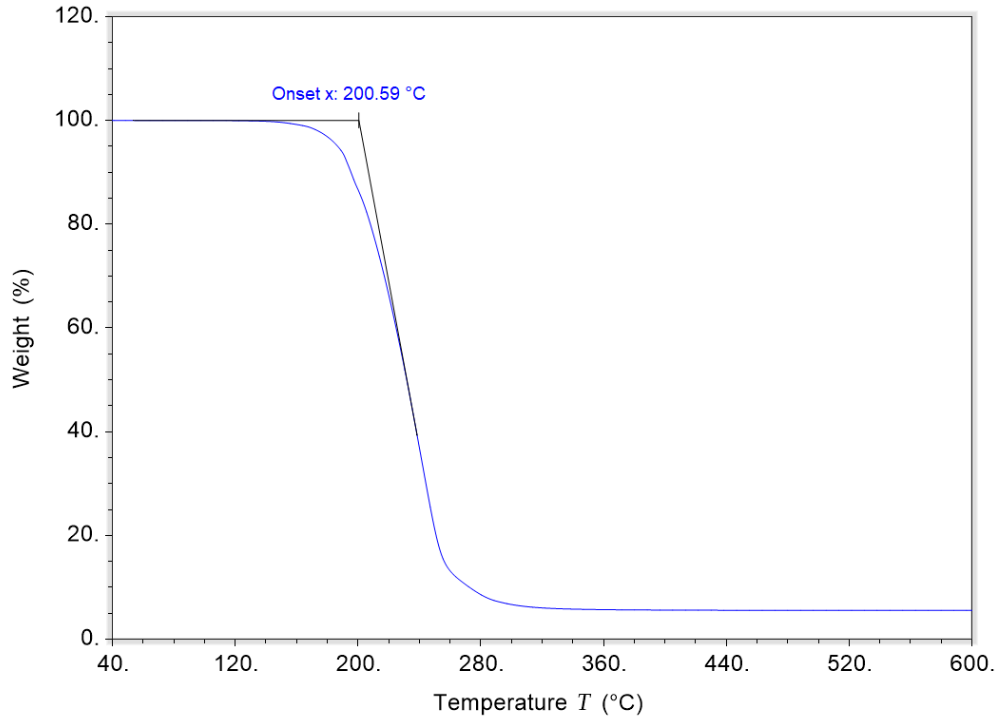

Figure S.28. TGA thermogram of octyldimethylglycinium indole-3-butyrate ***(IL1)***

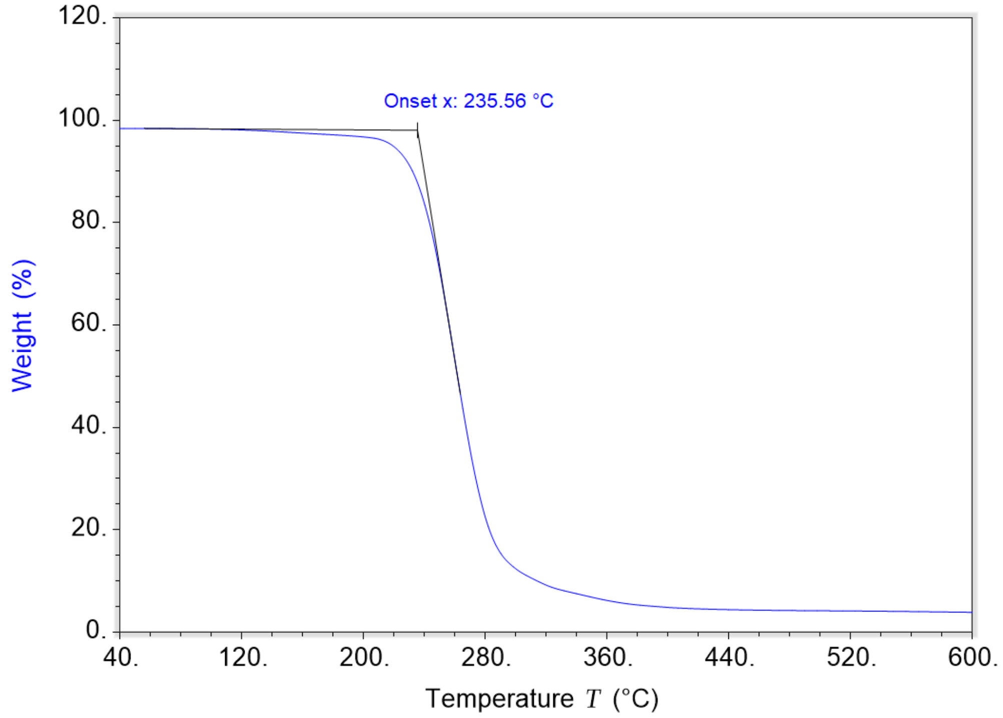

Figure S.29. TGA thermogram of decyldimethylglycinium indole-3-butyrate ***(IL2)***

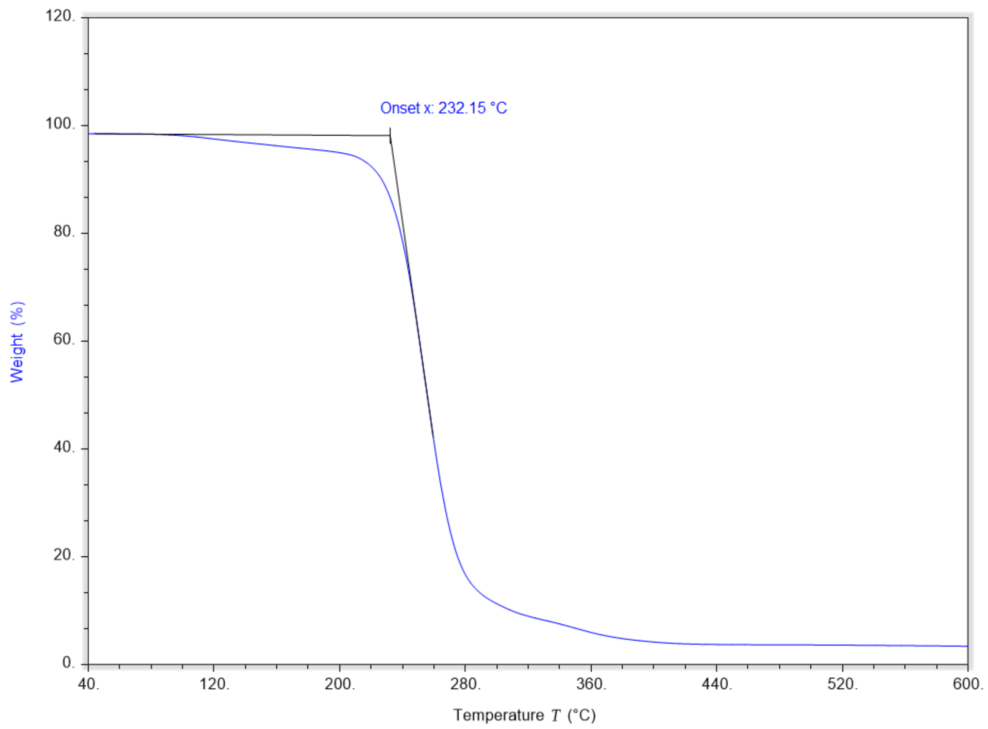

Figure S.30. TGA thermogram of dodecyldimethylglycinium indole-3-butyrate ***(IL3)***

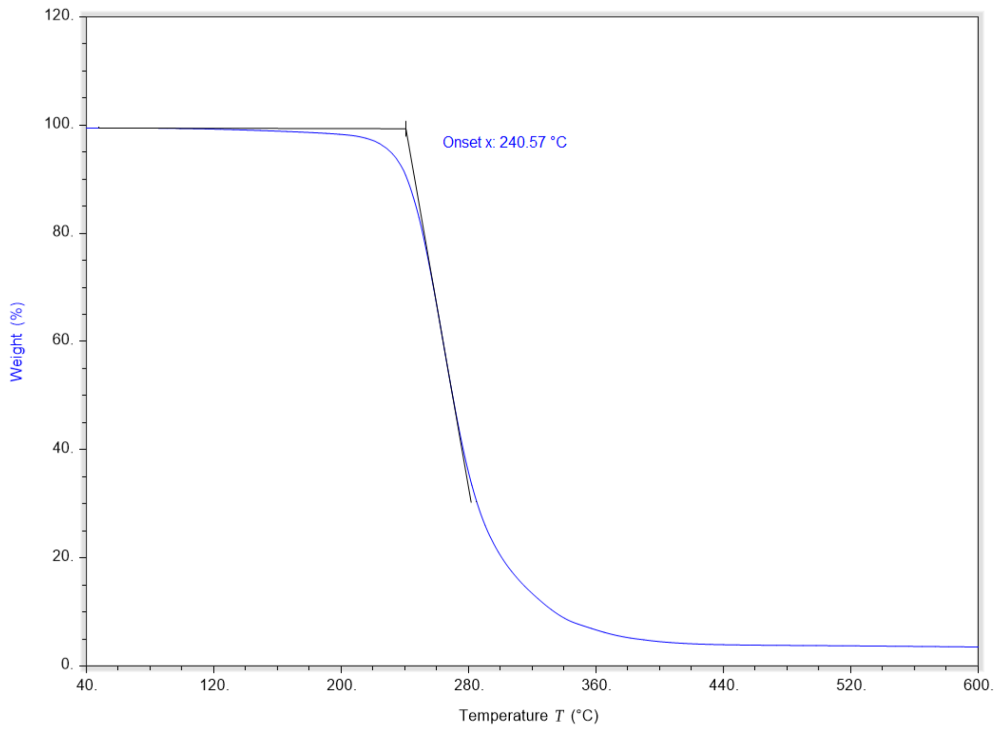

Table S1. Green Chemistry metrics determined for salts **1-3** and ILs **IL1-IL3**.

No.	Atom economy	Percentage yield	Reaction mass efficiency	Solvent intensity	Environmental factor
	[%]	[%]	[%]	[-]	[-]
**1**	77	96	74	8.53	0.31
**2**	79	97	69	8.44	0.31
**3**	81	98	65	8.36	0.30
**IL1**	85	96	81	5.86	0.19
**IL2**	86	95	76	5.92	0.19
**IL3**	86	97	74	5.80	0.18

Table S2. Effect of 25 ppm of IBA aqueous solutions of the **IL1-IL3** and the salts **1-3** on shoot and root length of white mustard. Control – control sample without the addition of salts or ILs.

No.	Lenght [cm]			
	root	±SE	shoot	±SE
**1**	0.70	0.10	3.50	0.39
**2**	2.67	0.24	4.70	0.42
**3**	2.66	0.29	4.82	0.45
**IL1**	3.55	0.28	8.32	0.61
**IL2**	3.78	0.22	7.67	0.42
**IL3**	3.05	0.39	6.41	0.46
**[K][IBA]**	2.91	0.21	4.73	0.11
**Control**	1.75	0.56	3.46	0.56

Table S3. Effect of 25 ppm of IBA aqueous solutions of the **IL1-IL3** and the salts **1-3** on shoot and root length of sorghum. Control – control sample without the addition of salts or ILs.

No.	Lenght [cm]			
	root	±SE	shoot	±SE
**1**	2.23	0.44	1.66	0.52
**2**	4.62	0.63	5.45	0.71
**3**	5.24	0.62	6.01	0.86
**IL1**	7.62	0.26	8.52	0.36
**IL2**	5.60	0.42	8.14	0.41
**IL3**	5.31	0.25	7.00	0.54
**[K][IBA]**	5.42	0.50	6.20	0.69
**Control**	5.20	0.62	4.73	0.68
